# An Alanine Aminotransferase Is Required for Biofilm-Specific Resistance of Aspergillus fumigatus to Echinocandin Treatment

**DOI:** 10.1128/mbio.02933-21

**Published:** 2022-03-07

**Authors:** Joshua D. Kerkaert, François Le Mauff, Benjamin R. Wucher, Sarah R. Beattie, Elisa M. Vesely, Donald C. Sheppard, Carey D. Nadell, Robert A. Cramer

**Affiliations:** a Department of Microbiology and Immunology, Geisel School of Medicine at Dartmouth, Hanover, New Hampshire, USA; b Department of Microbiology and Immunology, Faculty of Medicine, McGill University, Montreal, Quebec, Canada; c Infectious Disease and Immunity in Global Health, Research Institute of McGill University Health Center, Montreal, Quebec, Canada; d McGill Interdisciplinary Initiative in Infection and Immunity, Montreal, Quebec, Canada; e Department of Biological Sciences, Dartmouth College, Hanover, New Hampshire, USA; f Department of Pediatrics, Carver College of Medicine, University of Iowa, Iowa City, Iowa, USA; Institut Pasteur; Washington University School of Medicine

**Keywords:** *Aspergillus fumigatus*, biofilm, extracellular matrix, galactosaminogalactan, alanine, metabolism, echinocandins, antifungal drugs, biofilms, cell wall, echinocandin, hypoxia

## Abstract

Alanine metabolism has been suggested as an adaptation strategy to oxygen limitation in organisms ranging from plants to mammals. Within the pulmonary infection microenvironment, Aspergillus fumigatus forms biofilms with steep oxygen gradients defined by regions of oxygen limitation. An alanine aminotransferase, AlaA, was observed to function in alanine catabolism and is required for several aspects of A. fumigatus biofilm physiology. Loss of *alaA*, or its catalytic activity, results in decreased adherence of biofilms through a defect in the maturation of the extracellular matrix polysaccharide galactosaminogalactan (GAG). Additionally, exposure of cell wall polysaccharides is also impacted by loss of *alaA*, and loss of AlaA catalytic activity confers increased biofilm susceptibility to echinocandin treatment, which is correlated with enhanced fungicidal activity. The increase in echinocandin susceptibility is specific to biofilms, and chemical inhibition of *alaA* by the alanine aminotransferase inhibitor β-chloro-l-alanine is sufficient to sensitize A. fumigatus biofilms to echinocandin treatment. Finally, loss of *alaA* increases susceptibility of A. fumigatus to *in vivo* echinocandin treatment in a murine model of invasive pulmonary aspergillosis. Our results provide insight into the interplay of metabolism, biofilm formation, and antifungal drug resistance in A. fumigatus and describe a mechanism of increasing susceptibility of A. fumigatus biofilms to the echinocandin class of antifungal drugs.

## INTRODUCTION

Aspergillus fumigatus is a ubiquitous filamentous fungus with a prominent ecological role in the decomposition of organic carbon and is easily isolated from compost piles and similar environments (1). Within compost piles a complex set of microenvironments can emerge along temperature and nutrient gradients that naturally form as saprophytes become metabolically active (2, 3). Thus, A. fumigatus has evolved a significant degree of metabolic flexibility and thermotolerance (4, 5). However, these saprophytic fitness traits also increase the fungus’ pathogenic potential, leading to A. fumigatus being the causative agent of a variety of immune status-dependent human diseases (6–8), with the most lethal disease manifestation being invasive pulmonary aspergillosis (IPA).

IPA occurs primarily in individuals with a suppressed innate immune system, such as individuals undergoing solid-organ transplantation or chemotherapy (7, 9). Tragically, antifungal therapy options for IPA remain limited and are often ineffective, with recent clinical trials reporting 12-week mortality rates of 28 to 45% depending on therapeutic regimen and host immune status (10–13). One class of antifungal drugs, the echinocandins, inhibits synthesis of cell wall β-glucans and is better tolerated by patients than drugs belonging to the azole or polyene classes of antifungals. In many pathogenic yeasts, such as Candida albicans, echinocandin treatment has fungicidal activity and is utilized as a first-line treatment. While echinocandins yield some level of cell lysis when applied to A. fumigatus hyphae, these drugs are primarily fungistatic against A. fumigatus, as it is intrinsically tolerant to echinocandins and will exhibit residual growth even at high concentrations of drug (14). In many cases this tolerance results in a paradoxical phenomenon where the fungus will recover growth as the concentration of drug increases beyond a minimal effective concentration (MEC) (14–16). Thus, echinocandins have primarily been utilized as a salvage therapy for IPA, and strategies to increase their efficacy in treatment of IPA are potentially of great clinical significance.

Recent studies have established that A. fumigatus forms robust biofilms within the infection environment (17, 18). The hyphae within the A. fumigatus biofilm are coated with the extracellular matrix (ECM) polysaccharide galactosaminogalactan (GAG), which is composed of a heterogenous mixture of galactose and N-acetylgalactosamine. GAG functions as a primary adherence factor for A. fumigatus biofilms as well as an immunomodulatory compound (19–22). After synthesis, GAG requires partial deacetylation via the Agd3 deacetylase to function in both capacities, and strains lacking the ability to either produce GAG or deacetylate GAG are unable to adhere to surfaces (23–25). While some transcriptional regulatory machinery surrounding GAG biosynthesis and maturation has been described, mechanisms underlying biofilm formation and ECM regulation remain to be fully defined (19, 26).

Insights into A. fumigatus biofilm formation are of great importance, as these biofilms have been demonstrated to display clinically relevant emergent properties, including increased resistance to antifungal drugs (27–29). A major factor contributing to increased drug resistance is the formation of oxygen-limited, hypoxic microenvironments within the biofilm (27, 30). These same hypoxic microenvironments have been observed to exist in the infection environment, and the ability to adapt to oxygen limitation is essential for disease progression and full virulence (31, 32). While some transcriptional regulators of A. fumigatus oxygen adaptation have been identified (32–34), how the fungus metabolically adapts to low oxygen and how these metabolic pathways go on to impact broader A. fumigatus physiology remain to be fully appreciated.

Alanine metabolism has been associated with adaptation to oxygen limitation in numerous organisms, ranging from plant roots adapting to waterlogging (35, 36) to exercise-induced oxygen deprivation in muscle cells (37). Analysis of published transcriptomics data sets consistently show large increases in the mRNA levels of an alanine aminotransferase, here termed *alaA*, upon exposure of A. fumigatus to an oxygen-limiting environment (17, 34, 38–40). Additionally, alanine is one of only a few amino acids detectable in human bronchoalveolar lavage (BAL) fluid and bronchial wash (BW) fluid, indicating it is readily available in the airway environment (41) and may serve as a carbon or nitrogen source for A. fumigatus.

**FIG 1:**
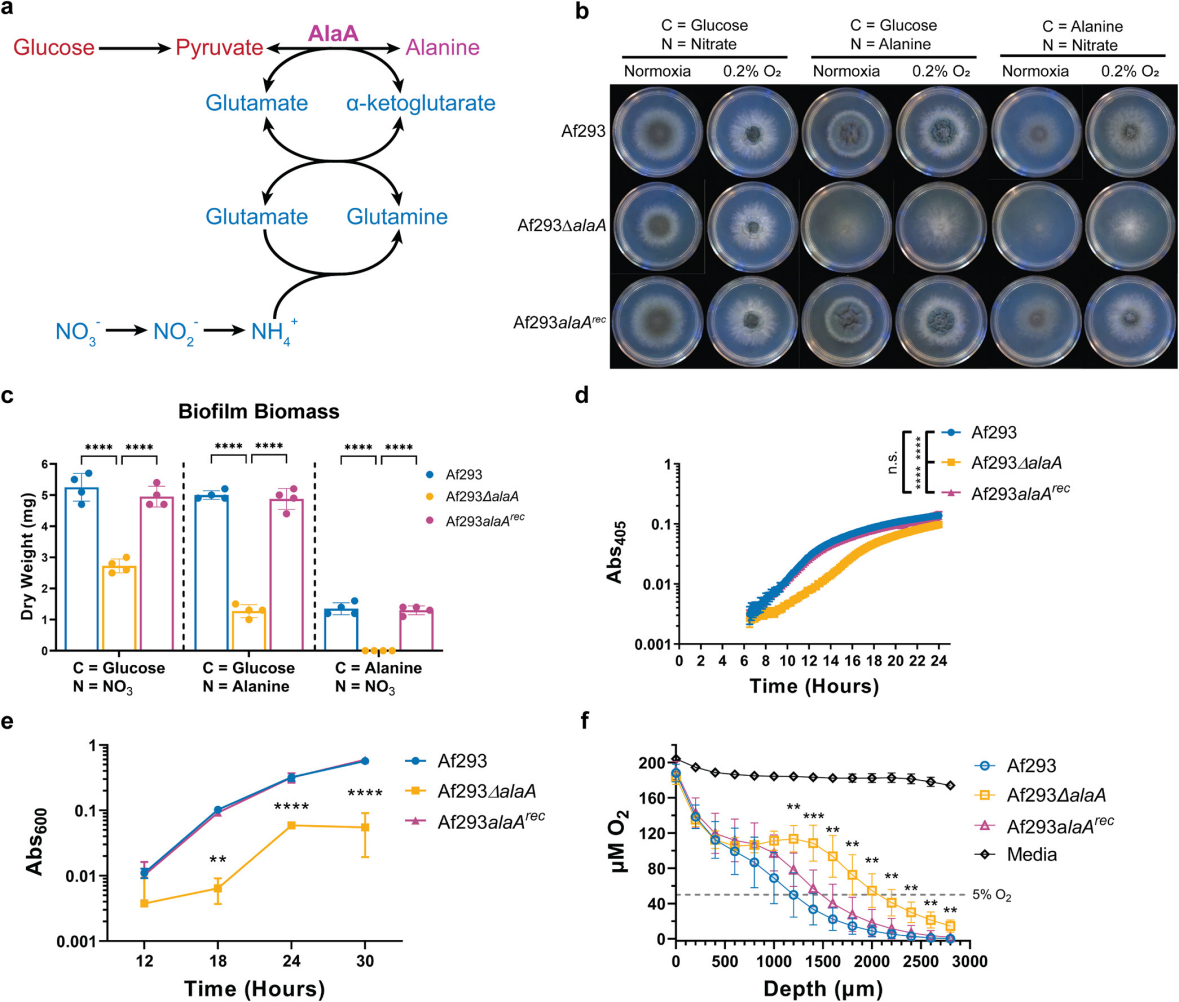
An alanine aminotransferase is required for alanine catabolism and normal biofilm physiology. (A) Reaction catalyzed by AlaA and its position in central carbon and nitrogen metabolism. (B) Growth of Af293Δ*alaA* strain on minimal medium containing the indicated sole carbon and nitrogen sources in ambient oxygen (normoxia) and 0.2% oxygen environments. Images are representative of four replicate cultures. (C) Dry biomass of biofilms grown in minimal medium containing the indicated sole carbon and nitrogen sources for 24 h (*n* = 4). Each replicate is shown along with the means ± standard deviations (SD). (D) Representative static growth assay of Af293Δ*alaA* strain over 24 h of biofilm growth (*n* = 6 technical replicates). The experiment was repeated at least three times with similar results. (E) Crystal violet adherence assay of biofilms grown for 12, 18, 24, and 30 h (*n* = 3). (F) Oxygen concentration as a function of distance from the air-liquid interface in 24-h biofilms (*n* ≥ 7). Culture volumes are approximately 3,000 μm in depth. **, *P* < 0.01; ***, *P* < 0.001; ****, *P* < 0.0001; ns, not significant by either two-way analysis of variance (ANOVA) with a Tukey’s multiple-comparison test (C, E, and F) or one-way ANOVA with a Tukey’s multiple-comparison test (D). All graphs show the means ± SD unless otherwise stated.

Here, we explore the role of A. fumigatus alanine metabolism via the alanine aminotransferase AlaA. Alanine aminotransferases catalyze the interconversion of pyruvate and alanine, utilizing glutamate as an amino group donor and, thus, participates in both carbon and nitrogen metabolism (Fig. 1A). While we observe that AlaA-mediated metabolic reactions are not essential for low oxygen growth, AlaA catalytic activity is critical for normal biofilm physiology where oxygen gradients naturally form. Moreover, AlaA is crucial for growth and full fitness when alanine is the sole carbon or nitrogen source. Unexpectedly, *alaA* is required for maturation of the ECM polysaccharide GAG and exposure of cell wall polysaccharides. Loss or inhibition of AlaA results in a striking reduction of echinocandin resistance in A. fumigatus biofilms both *in vitro* and *in vivo*.

## RESULTS

### The alanine aminotransferase *alaA* is required for efficient catabolism of l-alanine.

To assess the role of *alaA* in A. fumigatus metabolism and stress resistance, strains lacking the open reading frame of the *alaA* gene were generated in the Af293 (Af293Δ*alaA*) and CEA10 (also called CBS144.89) (CEA10Δ*alaA*) backgrounds along with respective reconstituted strains in which *alaA* was ectopically reintroduced into the Af293Δ*alaA* and CEA10Δ*alaA* genomes under the control of its native promoter (Af293*alaA^rec^* and CEA10*alaA^rec^* strains). Both Af293Δ*alaA* and CEA10Δ*alaA* strains grew on solid glucose minimal medium (GMM), where glucose is the sole carbon source and nitrate is the sole nitrogen source, indicating that sufficient alanine was generated for growth independent of *alaA* under these *in vitro* conditions (Fig. 1B; see also Fig. S1A in the supplemental material). However, radial growth of the *alaA* null strains on solid GMM was approximately 10% less than that of their respective wild-type (WT) and reconstituted strains at both ambient O_2_ and 0.2% O_2_, indicating a role for this protein in fungal metabolism in the presence of its preferred carbon source (Fig. 1B and Fig. S1A). When the *alaA* null strains were grown on solid medium with l-alanine as the sole carbon or sole nitrogen source, the strains displayed severe growth defects (Fig. 1B and Fig. S1A). Surprisingly, both the WT and *alaA* null strains grew more robustly at 0.2% O_2_ than ambient O_2_ when alanine was the sole carbon or nitrogen source, despite alanine being a nonfermentable carbon source (Fig. 1B and Fig. S1A). These data suggest that *alaA* plays an important role in alanine catabolism in A. fumigatus colony biofilms, with glucose as the sole carbon source. While *alaA* may play a minor role in alanine biosynthesis, as evidenced by the 10% growth reduction, it is likely that an alternative pathway is utilized as the primary means of alanine biosynthesis. Specifically, the A. fumigatus genome encodes a putative alanine-glyoxylate aminotransferase (Afu1g09470), which generates alanine from pyruvate using glycine as an amino-group donor, and recent evidence in Saccharomyces cerevisiae suggests fungal GABA aminotransferase can generate alanine using GABA as the amino-group donor (42). Moreover, under low-oxygen conditions, an alternative pathway for alanine catabolism is clearly present that promotes growth in the absence of AlaA. Thus, *alaA* plays an important role in A. fumigatus metabolism in multiple carbon, nitrogen, and oxygen environments.

10.1128/mBio.02933-21.1 FIG S1 *alaA* is required for alanine catabolism and biofilm physiology in the CEA10 strain background. (A) Growth of CEA10Δ*alaA* strain on minimal medium containing the indicated sole carbon and nitrogen sources in ambient oxygen and 0.2% oxygen environments. (B) Static growth assay of CEA10Δ*alaA* strain over the first 24 h of biofilm growth. Mean ± SD from 6 technical replicates is shown. Experiment was repeated a minimum of 3 times with similar results. *, *P* < 0.05; ***, *P* < 0.001 by one-way ANOVA with a Tukey’s multiple-comparison test. (C) Crystal violet adherence assay of 24-h biofilms (*n* = 6). ****, *P* < 0.0001; ns, not significant as determined by one-way ANOVA with a Tukey’s multiple comparisons test. (D and E) Conidial viability assay. Conidia were plated using the top-agar method at a known concentration and colony-forming units (CFU) were determined (*n* = 4). ns, not significant as determined by one-way ANOVA with a Tukey’s multiple-comparison test. Download FIG S1, TIF file, 2.6 MB. Copyright © 2022 Kerkaert et al. 2022 Kerkaert et al. https://creativecommons.org/licenses/by/4.0/ This content is distributed under the terms of the Creative Commons Attribution 4.0 International license.

### Robust adherence and growth of A. fumigatus biofilms is dependent on *alaA*.

A. fumigatus submerged biofilms naturally become increasingly oxygen deprived as they mature (27, 30). Thus, we next investigated the role of *alaA* in an A. fumigatus submerged biofilm model. To assess biofilm formation in GMM and further quantify the role of AlaA in alanine metabolism, we quantified the dry biomass of submerged biofilms grown for 24 h in GMM and with alanine as a sole carbon or nitrogen source. Loss of *alaA* resulted in a 40 to 50% decrease in submerged biofilm biomass in GMM. This growth defect was exacerbated when alanine was the sole carbon or nitrogen source, with no biomass recovered when alanine was the sole nitrogen source (Fig. 1C). These data further support a catabolic role for AlaA. Additionally, we utilized a static growth assay to assess biofilm growth kinetics, which revealed that *alaA* null strains had a longer lag phase than their respective WT or reconstituted strains, which could be due to either decreased conidial viability or a delay in germination (Fig. 1D and Fig. S1B). A conidial viability assay revealed no difference in the viability of dormant conidia between the WT, *alaA* null, and reconstituted strains, indicating the extended lag phase is due to a delay in conidial germination in the submerged biofilm model (Fig. S1D and E). To further determine if *alaA* had broad physiological impacts on A. fumigatus submerged biofilm formation, a crystal violet adherence assay was utilized to quantify adherence of the *alaA* null biofilms to abiotic surfaces. To account for any impacts of the germination delay on biofilm formation, the adherence of Af293Δ*alaA* strain was measured over a time course from an immature biofilm at 12 h to a highly mature biofilm at 30 h. At all time points after 12 h, Af293Δ*alaA* strain had a severe defect in adherence compared to the WT and reconstituted strains (Fig. 1E). The CEA10Δ*alaA* strain was also tested for adherence and showed a similar inability to strongly adhere to surfaces (Fig. S1C). Finally, we quantified oxygen levels within 24-h biofilm cultures of Af293Δ*alaA* strain. The *alaA* null strain cultures were significantly more oxygenated than the WT and reconstituted strain biofilms (Fig. 1F). However, the portion of the culture containing the bulk of the biofilm’s biomass, at a depth of ∼2,000 μm to 3,000 μm based on previous microscopy studies (27), was still below 5% O_2_ and likely experiencing hypoxia. Therefore, while the loss of *alaA* has an impact on colony biofilm growth on solid media, *alaA* appears to play a greater role in A. fumigatus submerged biofilm physiology, where steep oxygen and nutrient gradients naturally occur (27).

### Catalytic activity of AlaA is required for adherence and alanine growth but not mitochondrial localization.

**FIG 2:**
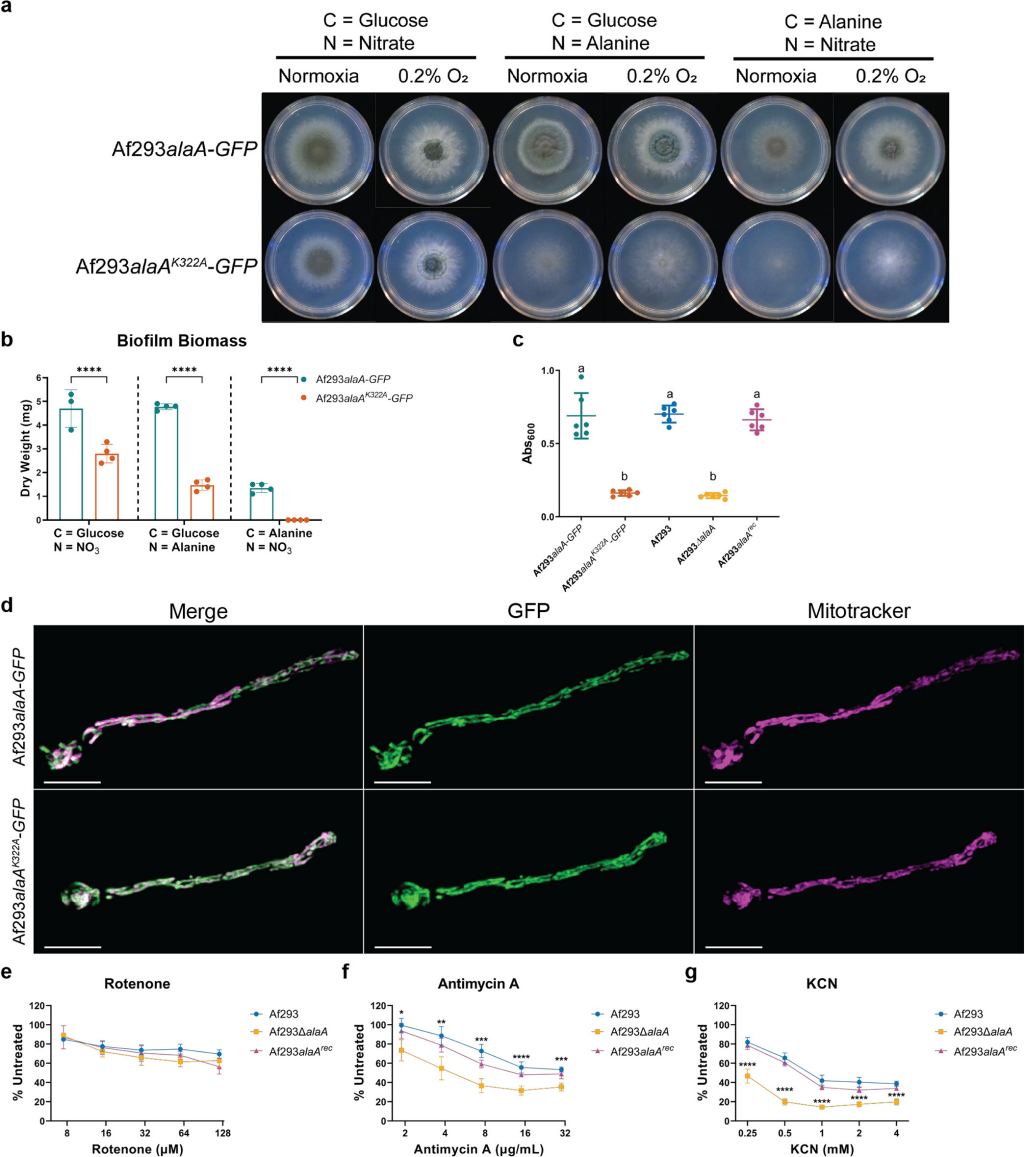
Catalytic activity of AlaA is required for alanine catabolism, adherence of biofilms, and mitochondrial function. (A) Growth of Af293*alaA^K322A^-GFP* strain on minimal medium containing the indicated sole carbon and nitrogen sources in ambient oxygen (normoxia) and 0.2% oxygen environments. Images are representative of four replicate cultures. (B) Dry biomass of biofilms grown in minimal medium containing the indicated sole carbon and nitrogen sources (*n* ≥ 3). Each replicate along with the means ± SD are shown. ****, *P* < 0.0001 as determined by two-way ANOVA with a Tukey’s multiple-comparison test. (C) Crystal violet adherence assay of 24-h biofilms (*n* = 6). Each replicate along with the means ± SD are shown. a versus b, *P* < 0.0001 in all comparisons as determined by one-way ANOVA with a Tukey’s multiple-comparison test. (D) Representative micrographs of germlings containing C-terminal GFP-tagged AlaA alleles (green) stained with MitoTracker Deep Red FM (magenta). (E to G) Twenty-four-hour biofilms were established in the absence of drug and treated with rotenone (E), antimycin A (F), or KCN (G) at the indicated concentrations for 3 h, and viability was determined by XTT assay. Means ± SD are shown for *n* = 4 for each experiment. *, *P* < 0.05; **, *P* < 0.01; ***, *P* < 0.001; ****, *P* < 0.0001 as determined by two-way ANOVA with a Tukey’s multiple-comparison test. The highest *P* value for Af293Δ*alaA* strain compared to both Af293 and Af293*alaA^rec^* strains is shown.

Recent studies in Saccharomyces cerevisiae have determined Alt1, the AlaA homolog, has moonlighting functions, or functions independent of an enzyme’s catalytic activity (42, 43). To test if AlaA was functioning through its catalytic activity, we generated a catalytically inactive allele of *alaA* by changing the conserved catalytic lysine residue at position 322 to an alanine and adding a C-terminal green fluorescent protein (GFP) tag (Fig. S2A) (44). This construct was then introduced into the native locus of *alaA* in the Af293 background (Af293*alaA^K322A^-GFP*). Additionally, the WT allele was modified with a C-terminal GFP tag and was transformed in the same manner (Af293*alaA-GFP*). Af293*alaA-GFP* and Af293*alaA^K322A^-GFP* strains grew on alanine as the sole carbon or sole nitrogen source in a manner similar to that of the WT and *alaA* null strains, respectively, confirming that catalytic function was abolished and that the GFP tag did not interfere with protein function (Fig. 2A and B). A crystal violet adherence assay revealed that Af293*alaA^K322A^-GFP* strain exhibited an adherence defect equivalent to the deletion of the entire *alaA* gene (Fig. 2C). Confocal microscopy of these two strains in combination with MitoTracker Deep Red FM revealed that both the WT and catalytically inactive *alaA* alleles were stably expressed and localized to the mitochondria in planktonic hyphae (Fig. 2D and Fig. S2D). Therefore, AlaA catalytic activity is required for adherence and alanine catabolism but not mitochondrial localization.

10.1128/mBio.02933-21.2 FIG S2 AlaA protein domain architecture and degree of similarity to alanine aminotransferases of several model systems. (A) AlaA linear protein structure highlighting the aminotransferase class I/II domain, predicted PLP-binding residues, and the catalytic lysine residue mutated in the catalytic null strain (Af293*alaA^K322A^-GFP*). (B) Phylogeny of AlaA relative to human, murine, and Saccharomyces cerevisiae alanine aminotransferases. All species except A. fumigatus have two alanine aminotransferases encoded by their genomes. Proteins were aligned in MEGA X using MUSCLE and a maximum-likelihood tree was generated. Scale bar and branch lengths refer to substitutions per site. (C) Percent identity matrix of the alanine aminotransferases shown in panel B. (D) Representative micrograph of wild-type Af293 germlings stained with MitoTracker Deep Red FM (magenta). No fluorescence was observed in the GFP channel. Download FIG S2, TIF file, 1.0 MB. Copyright © 2022 Kerkaert et al. 2022 Kerkaert et al. https://creativecommons.org/licenses/by/4.0/ This content is distributed under the terms of the Creative Commons Attribution 4.0 International license.

### Mitochondrial function is impacted by *alaA*.

In mammals it has been demonstrated that the localization of an alanine aminotransferase is predictive of whether it primarily serves a catabolic or anabolic function (45). In mammals as well as S. cerevisiae, mitochondrially localized alanine aminotransferases have been demonstrated to have a primarily catabolic role and impact mitochondrial function (42, 45, 46). The growth characteristics of *alaA*-deficient strains suggest a primarily catabolic role for the enzyme (Fig. 1B and C, 2A and B, and Fig. S1A); thus, we sought to address if *alaA* was also involved in mitochondrial function. To test this hypothesis, biofilms of Af293, Af293Δ*alaA*, and Af293*alaA^rec^* strains were examined for sensitivity to the complex I inhibitor rotenone, the complex III inhibitor antimycin A, and the complex IV inhibitor KCN. Biofilms were grown to maturity (24 h) prior to application of the mitochondrial inhibitor for 3 h. Metabolic activity was then compared to that of untreated biofilms via reduction of 2,3-bis-(2-methoxy-4-nitro-5-sulfophenyl)-2H-tetrazolium-5-carboxanilide salt (XTT). Af293Δ*alaA* biofilms were more susceptible to damage by antimycin A and KCN but not rotenone. This indicates that *alaA* is required for proper functioning of the later portion of the electron transport chain.

### Loss of *alaA* leads to alterations in the adherence-mediating polysaccharide GAG.

**FIG 3:**
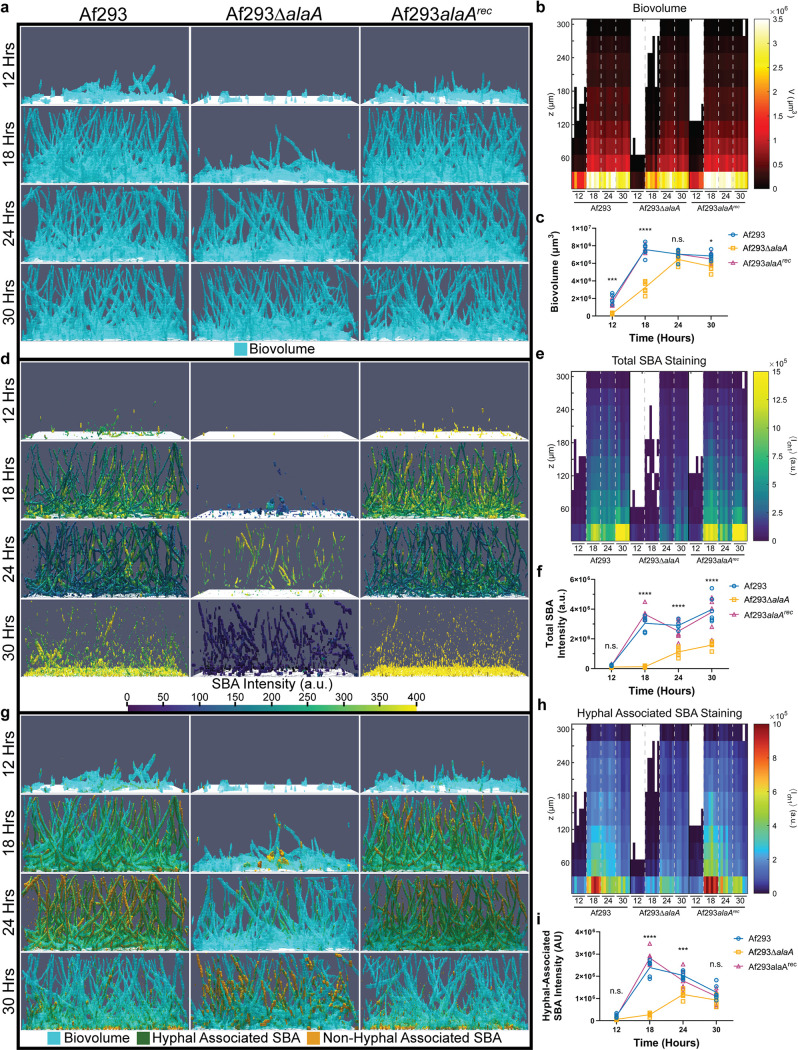
Loss of *alaA* alters extracellular matrix staining by the galactosaminogalactan binding lectin SBA. (A) Representative image renderings of biovolume in the first 300 μm of biofilms grown for 12, 18, 24, and 30 h. Biofilms were stained with calcofluor white and FITC-SBA, followed by fixing with paraformaldehyde. Biovolume was determined by segmentation of the calcofluor white stain of each image. (B) Heatmap of biovolume as a function of height from the base of the biofilm. (C) Global segmented biovolume quantifications of each biofilm. (D) Representative image renderings of FITC-SBA staining intensity corresponding to biomass images in panel A. Renderings show FITC-SBA matrix intensity mapped onto the segmented FITC-SBA stain. a.u., arbitrary units. (E) Heatmap of FITC-SBA intensity as a function of height from the base of the biofilm. (F) Sum intensity quantification of FITC-SBA staining for each biofilm. (G) Representative merged image renderings of the segmented biovolume (calcofluor white), shown in blue, and segmented FITC-SBA stain, shown in orange. Hyphal associated SBA staining will appear green as a result of the overlap between the two channels. SBA was considered hyphal associated or non-hyphal associated based on overlap in the segmented biomass. (H) Heatmap of hyphal associated FITC-SBA intensity as a function of height from the base of the biofilm. (I) Sum intensity quantification of hyphal associated FITC-SBA staining for each biofilm. Each graph and heatmap shows the individual replicates for each time point (*n* = 6). For panels C, F, and I, the line goes through the mean of each time point. *, *P* < 0.05; ***, *P* < 0.001; ****, *P* < 0.0001; ns, not significant as determined by two-way ANOVA with a Tukey’s multiple comparison test (C, F, and I).

The primary adherence factor for A. fumigatus submerged biofilms studied to date is the extracellular matrix polysaccharide galactosaminogalactan (GAG) (19, 20, 23), and the lack of adherence observed in the *alaA* null strain suggests a role in GAG production or maturation. To examine GAG production, we first utilized a fluorescently labeled lectin specific to *N*-acetyl-d-galactosamine (GalNAc) residues, found in the GAG polysaccharide, fluorescein isothiocyanate-soybean agglutinin (FITC-SBA). Biofilms of Af293, Af293Δ*alaA*, and Af293*alaA^rec^* strains were stained with SBA to visualize GAG and calcofluor white, which binds chitin, to visualize biomass at 12, 18, 24, and 30 h of growth (Fig. 3A, D, and G). Spinning-disk confocal microscopy was utilized to image the first 300 μm of the biofilms, followed by quantification using BiofilmQ (47). As seen in growth curve experiments (Fig. 1F), Af293Δ*alaA* strain had a lower biovolume at 12 and 18 h (Fig. 3A to C). Total SBA staining of the GAG polysaccharide was quantified as the sum intensity of the SBA stain in each image, revealing that Af293Δ*alaA* biofilms had less total SBA staining than the WT and reconstituted strains starting at 18 h of growth (Fig. 3D to F). While the SBA staining tightly associated with the cell wall at all time points in the Af293Δ*alaA* strain, at 30 h in the WT and reconstituted strains the SBA staining pattern shifted from hyphal associated to primarily staining the extracellular milieu (Fig. 3D and G). We quantified the hyphal associated SBA staining as the sum intensity of SBA stain that overlapped with the segmented calcofluor white stain, thereby showing GAG in relation only to hyphal biovolume. In the WT and reconstituted strain biofilms, hyphal associated SBA peaked at 18 h and decreased at 24 and 30 h as matrix was shed from the hyphae into the extracellular milieu (Fig. 3G to I). This was in contrast to total SBA staining, which remained relatively consistent from 18 to 30 h of growth in the WT and reconstituted strains (Fig. 3E).

**FIG 4:**
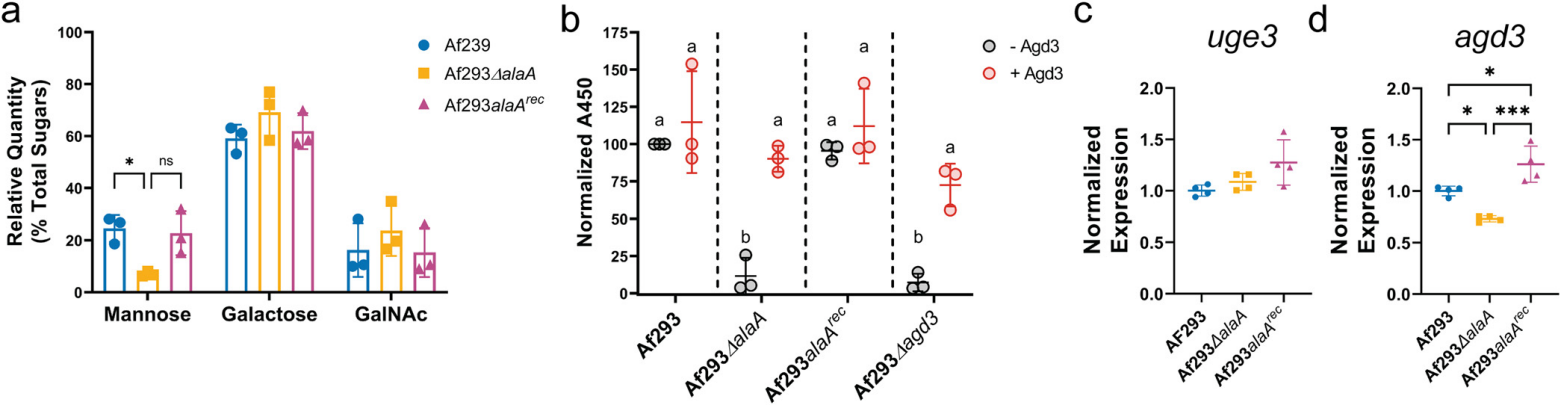
*alaA* is required for proper deacetylation of galactosaminogalactan. (A) Monosaccharide analysis of extracellular matrix polysaccharides (*n* = 3). *, *P* < 0.05; ns, not significant by two-way ANOVA with a Tukey’s multiple-comparison test. (B) Enzyme-linked lectin assay (ELLA) of biofilm extracellular matrix left untreated or treated with recombinant Agd3. a versus b, *P* < 0.05 for all comparisons as determined by two-way ANOVA with a Tukey’s multiple-comparison test. (C and D) Expression of *uge3* (C) and *agd3* (D) in 24-h biofilm cultures as determined by RT-qPCR. *, *P* < 0.05; ***, *P* < 0.001 as determined by one-way ANOVA with a Tukey’s multiple-comparison test for panels C and D. For all graphs, each replicate along with the means ± SD are shown.

To chemically define how GAG was being altered in the *alaA* null strain, monosaccharide analysis of ECM polysaccharides and an enzyme-linked lectin assay (ELLA) were conducted. Monosaccharide analysis revealed that the *alaA* null strain’s ECM polysaccharide composition was similar to that of the WT strain, with a trend toward decreased relative levels of mannose, which is reflective of galactomannan levels (Fig. 4A). This finding suggests that the altered ECM is primarily due to a change in the maturation of the ECM polysaccharides rather than a difference in the base polysaccharides produced. After GAG has been synthesized, partial deacetylation by the Agd3 deacetylase is necessary for functional adherence (23, 24). To test if GAG maturation was altered, we utilized an ELLA in combination with treatment of the ECM by recombinant Agd3. In principle, deacetylated GAG in supernatants allows for adherence to the walls of a polystyrene plate, whereas fully acetylated GAG cannot adhere and is easily removed by washing. Adherent, and therefore deacetylated, GAG can then be quantified by binding of a biotinylated SBA lectin coupled to a streptavidin-conjugated horseradish peroxidase. Additionally, the presence of fully acetylated GAG can be detected by pretreating samples with recombinant Agd3, producing deacetylated, adherent GAG that can then be detected by SBA. A strain lacking the *agd3* gene, which only produces fully acetylated GAG (23), was utilized as a control. The *alaA* and *agd3* null strains both yielded low levels of adherent (deacetylated) GAG compared to the WT, and this was rescued by treatment of ECM with recombinant Agd3 protein (Fig. 4B). Therefore, *alaA* is not required for GAG production but rather is required for deacetylation and maturation of the GAG polysaccharide into its functional form. Finally, mRNA abundance of *uge3* and *agd3* was measured from RNA isolated from 24-h biofilms of Af293, Af293Δ*alaA*, and Af293*alaA^rec^* strains to begin to distinguish if the observed differences in GAG are through a transcriptional or posttranscriptional mechanism of regulation. No differences in expression of *uge3* were observed (Fig. 4C). While a statistically significant decrease of ∼20% in *agd3* mRNA levels was observed (Fig. 4D), it is unclear if that level of mRNA difference could cause the degree of altered GAG deacetylation observed. Thus, while loss of *alaA* has a modest impact on *agd3* at the transcriptional level, the impact of *alaA* on GAG maturation is likely to be primarily posttranscriptional.

### Deletion of *alaA* leads to cell wall changes and increased susceptibility of biofilms to echinocandins.

**FIG 5:**
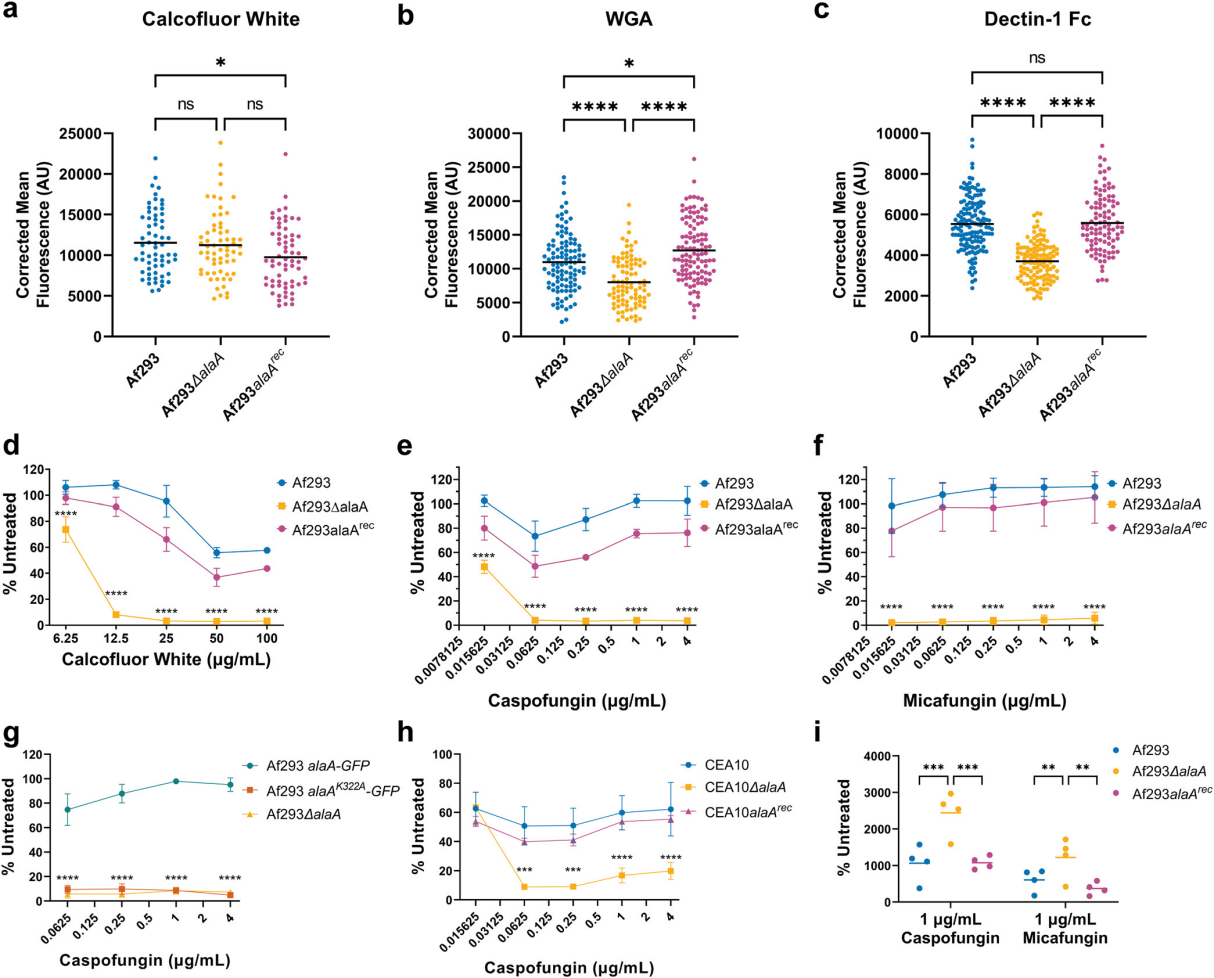
Loss of *alaA* leads to cell wall changes and increased susceptibility of biofilms to echinocandins. Germlings were stained with calcofluor white to quantify total chitin content (A), FITC-wheat germ agglutinin (WGA) to quantify surface exposed chitin (B), and Dectin-1 Fc to quantify surface-exposed β-glucans (C). Each data point represents an individual germling across three independent cultures per strain for each cell wall stain, and the lines correspond to the means. *, *P* < 0.05; ****, *P* < 0.0001; ns, not significant as determined by one-way ANOVA with a Tukey’s multiple-comparison test. (D to F) Twenty-four-hour biofilms were established in the absence of drug and treated with calcofluor white (D), caspofungin (E), or micafungin (F) at the indicated concentrations for 3 h, and viability was determined by XTT assay. Means ± SD are shown for *n* ≥ 3 experiments. ****, *P* < 0.0001 as determined by two-way ANOVA with a Tukey’s multiple-comparison test. The highest *P* value for Af293Δ*alaA* strain compared to both Af293 and Af293*alaA^rec^* strains is shown. (G) Af293*alaA^K322A^-GFP* biofilms were grown for 24 h and treated with caspofungin at the indicated concentrations for 3 h, and viability was determined by XTT assay. Means ± SD are shown for *n* = 3 replicates. ****, *P* < 0.0001 as determined by two-way ANOVA with a Tukey’s multiple-comparison test. The highest *P* values for Af293*alaA^K322A^-GFP* strain and Af293Δ*alaA* strain compared to Af293*alaA-GFP* strain are shown. No significant difference was observed between Af293*alaA^K322A^-GFP* strain and Af293Δ*alaA* strain. (H) CEA10Δ*alaA* biofilms were grown for 24 h and treated with caspofungin at the indicated concentrations for 3 h, and viability was determined by XTT assay. Means ± SD are shown for *n* = 3 replicates. ***, *P* < 0.001; ****, *P* < 0.0001 as determined by two-way ANOVA with a Tukey’s multiple-comparison test. The highest *P* values for CEA10Δ*alaA* strain compared to both CEA10 and CEA10*alaA^rec^* strains are shown. (I) Adenylate kinase release assay as a quantification of cell lysis. Twenty-four-hour biofilms were treated with 1 μg/mL caspofungin (left) or micafungin (right) for 3 h, and supernatant adenylate kinase activity was quantified. Each replicate and the means are shown (*n* = 4). **, *P* < 0.01; ***, *P* < 0.001 as determined by two-way ANOVA with a Tukey’s multiple-comparison test.

Given that GAG maturation was substantially impacted by loss of *alaA* and that *alaA* plays a role in metabolism, we asked if loss of *alaA* impacts additional cell wall polysaccharides. To test this hypothesis, germlings of Af293, Af293Δ*alaA*, and Af293*alaA^rec^* strains were stained with calcofluor white (to measure total chitin), wheat-germ agglutinin (WGA) (to measure surface exposed chitin), and soluble Dectin-1 Fc (to measure surface exposed β-glucans). Curiously, Af293Δ*alaA* germlings had lower WGA staining (exposed chitin) despite no difference in calcofluor white staining (total chitin) (Fig. 5A and B). Loss of *alaA* also decreased exposure of the immunostimulatory β-glucan polysaccharide, as determined by Dectin-1 Fc staining (Fig. 5C). These results were surprising, as it has been demonstrated that GAG masks β-glucans, and perturbations to GAG synthesis (19) or maturation (23) normally result in higher levels of Dectin-1 Fc staining. Together, these findings suggest Af293Δ*alaA* strain has lower levels of total cell wall β-glucans and that *alaA* is required for WT cell wall organization.

To determine if these cell wall changes translated to functional phenotypes, biofilms of Af293, Af293Δ*alaA*, and Af293*alaA^rec^* strains were tested for sensitivity to the cell wall-perturbing agent calcofluor white and the echinocandin class of antifungal drugs. Biofilms were grown to maturity (24 h) prior to application of cell wall stress for 3 h. Metabolic activity was then compared to that of untreated biofilms via reduction of XTT. Af293Δ*alaA* biofilms were significantly more susceptible to damage by calcofluor white at all concentrations tested, with greater than 90% inhibition beginning at 12.5 μg/mL (Fig. 5D). In contrast, the WT and reconstituted strains maintained at least 30% metabolic activity at even the highest concentration tested (100 μg/mL) (Fig. 5D). We next tested the strains for susceptibility to the echinocandin caspofungin. The WT and reconstituted biofilms displayed minimal damage regardless of concentration. Quantification also reveals signs of the paradoxical effect in the submerged biofilm model, where the fungus will recover growth as the concentration of drug increases beyond a MEC (Fig. 5E). Thus, similar to MEC and agar colony biofilm plate assays that begin with conidia, mature WT A. fumigatus biofilms are caspofungin tolerant. However, *alaA* null biofilms were highly susceptible to caspofungin and reached >90% inhibition at a concentration of 0.0625 μg/mL. Unlike WT biofilms, the *alaA* null biofilms did not display evidence of a paradoxical effect, with increasing concentrations of caspofungin yielding equivalent or greater damage (Fig. 5E).

To test if this increased susceptibility to caspofungin extended to other echinocandins, these experiments were validated with another echinocandin, micafungin. Similar to caspofungin treatment, the biofilms of WT and reconstituted strains were highly resistant to treatment with micafungin, whereas Af293Δ*alaA* strain was inhibited >90% at even the lowest concentration of drug tested, 0.015625 μg/mL (Fig. 5F). The catalytically inactive strain (Af293*alaA^K322A^*) was also tested for caspofungin sensitivity and displayed the same phenotype as Af293Δ*alaA* strain (Fig. 5G). Finally, to ensure this phenotype was not specific to the Af293 reference strain, these phenotypes were validated in another reference background, CEA10. CEA10, CEA10Δ*alaA*, and CEA10*alaA^rec^* biofilms were tested for susceptibility to caspofungin and again the loss of *alaA* resulted in increased echinocandin susceptibility (Fig. 5H). Moreover, the increased susceptibility of the *alaA* null mutant was confirmed by measuring adenylate kinase release (48, 49). In brief, adenylate kinase is normally present in very low quantities extracellularly, and increased release of adenylate kinase is indicative of cell lysis (49). Treatment of Af293Δ*alaA* biofilms with caspofungin or micafungin resulted in a greater release of adenylate kinase into the supernatant, indicating that loss of *alaA* potentiates the fungicidal effects of echinocandins (Fig. 5I and Fig. S3). Thus, the presence and catalytic activity of AlaA are required for the high level of echinocandin resistance observed in phylogenetically diverse A. fumigatus biofilms.

10.1128/mBio.02933-21.3 FIG S3 XTT assay corresponding to the cultures used in the adenylate kinase release assay. Biofilms were grown for 24 h and treated with 1 μg/mL caspofungin (left) or micafungin (right) for 3 h. Supernatants were used to quantify adenylate kinase activity (Fig. 5I), and an XTT assay was performed to measure viability of biofilm biomass. Each replicate and mean are shown (*n* = 4). **, *P* < 0.01; ***, *P* < 0.001 as determined by two-way ANOVA with a Tukey’s multiple-comparison test. Download FIG S3, TIF file, 0.3 MB. Copyright © 2022 Kerkaert et al. 2022 Kerkaert et al. https://creativecommons.org/licenses/by/4.0/ This content is distributed under the terms of the Creative Commons Attribution 4.0 International license.

We next tested if the increased susceptibility of Af293Δ*alaA* strain to echinocandins was biofilm specific or if it extended to more traditional measures of drug susceptibility that begin with exposing dormant conidia to the drug. Susceptibility to caspofungin was measured using radial growth of conidia on agar plates at three concentrations of caspofungin (0.25 μg/mL, 1 μg/mL, and 4 μg/mL). No significant differences in colony biofilm growth were observed between the strains at any concentration tested on agar surfaces (Fig. S4A to C). Additionally, the paradoxical effect was observed in all five strains tested, with increased growth as the concentration of caspofungin increased. Intriguingly, no difference in resistance to caspofungin was observed when a MEC assay was utilized with conidia of Af293, Af293Δ*alaA*, and Af293*alaA^rec^* strains (Fig. S4D). Therefore, the increased susceptibility of Af293Δ*alaA* strain to echinocandins is a biofilm-specific phenomenon, as no difference in susceptibility to caspofungin is observed when the drug is applied to dormant conidia.

10.1128/mBio.02933-21.4 FIG S4 Increased susceptibility of *alaA* null strains to caspofungin is biofilm specific. (A) Conidial radial growth assays of the indicated strains grown on the indicated concentrations of caspofungin in GMM for 72 h. Images are representative of four replicate cultures. (B) Quantification of radial growth as the diameter for each colony (*n* = 4). (C) Radial growth normalized to the untreated control for each strain. Individual replicates and mean are shown (*n* = 4) for panels B and C. (D) Minimum effective concentration (MEC) of caspofungin for the indicated strains at 24 and 48 h of incubation in GMM containing increasing concentrations of caspofungin (*n* = 3). Download FIG S4, TIF file, 2.7 MB. Copyright © 2022 Kerkaert et al. 2022 Kerkaert et al. https://creativecommons.org/licenses/by/4.0/ This content is distributed under the terms of the Creative Commons Attribution 4.0 International license.

### Deletion of *alaA* has strain- and biofilm-specific impacts on amphotericin B susceptibility.

The interaction between *alaA* and echinocandins led us to ask if the increased susceptibility of *alaA* null strain biofilms was specific to the echinocandins or if it extended to the polyene and azole classes of antifungals. Mature biofilms were treated with amphotericin B and voriconazole in the same manner as that described above, and the impact of each drug was assessed using XTT (Fig. S5A to F). Af293Δ*alaA* biofilms were significantly more susceptible to amphotericin B treatment than the WT and reconstituted strains, with a roughly 3- to 3.5-fold lower calculated IC_50_ (0.0827 μg/mL versus 0.286 μg/mL and 0.240 μg/mL, respectively) (Fig. S5A and C). In contrast, CEA10Δ*alaA* biofilms were not significantly more inhibited by amphotericin than its respective WT and reconstituted strains (Fig. S5B and D). This is the only phenotype observed thus far in which *alaA* had a different impact in the Af293 and CEA10 backgrounds, suggesting that *alaA* has both broad strain-independent and strain-specific impacts that are of clinical relevance. All biofilms were highly resistant to voriconazole treatment, and no significant differences were observed between the *alaA* null strains and their respective WT strains (Fig. S5E and F). Additionally, no difference in conidial susceptibility was observed for either amphotericin B or voriconazole utilizing MIC assays (Fig. S5G and H). Therefore, *alaA* does not appear to have a role in azole susceptibility but does have a biofilm- and strain-specific impact on amphotericin B susceptibility.

10.1128/mBio.02933-21.5 FIG S5 Amphotericin sensitivity of *alaA* null strains is both strain dependent and biofilm specific. (A to D) Susceptibility of 24-h Af293Δ*alaA* (A and C) and CEA10Δ*alaA* (B and D) biofilms to treatment with amphotericin B; 24-h biofilms were treated with the indicated concentrations of amphotericin for 3 h, and viability was assessed by XTT assay. (A and B) The mean ± SD is shown (*n* = 4) along with a nonlinear regression using a dose-response model (line) ± 95% confidence interval (shaded area). (C and D) Data from panels A and B were fit with a nonlinear dose response model, and the 50% inhibitory concentration (IC_50_) was calculated. The calculated IC_50_ ± the 95% confidence interval are displayed. (E and F) Susceptibility of 24-h Af293Δ*alaA* (E) and CEA10Δ*alaA* (F) biofilms to treatment with voriconazole. Biofilms were treated with the indicated concentrations of voriconazole for 3 h, and viability was assessed by XTT assay. The mean ± SD is displayed (*n* = 4). (G and H) MIC of conidia treated with increasing concentrations of amphotericin B (G) or voriconazole (H) for 24 and 48 h. Download FIG S5, TIF file, 0.9 MB. Copyright © 2022 Kerkaert et al. 2022 Kerkaert et al. https://creativecommons.org/licenses/by/4.0/ This content is distributed under the terms of the Creative Commons Attribution 4.0 International license.

### Chemical inhibition of AlaA by β-chloro-l-alanine decreases adherence and increases susceptibility of A. fumigatus biofilms to caspofungin.

**FIG 6:**
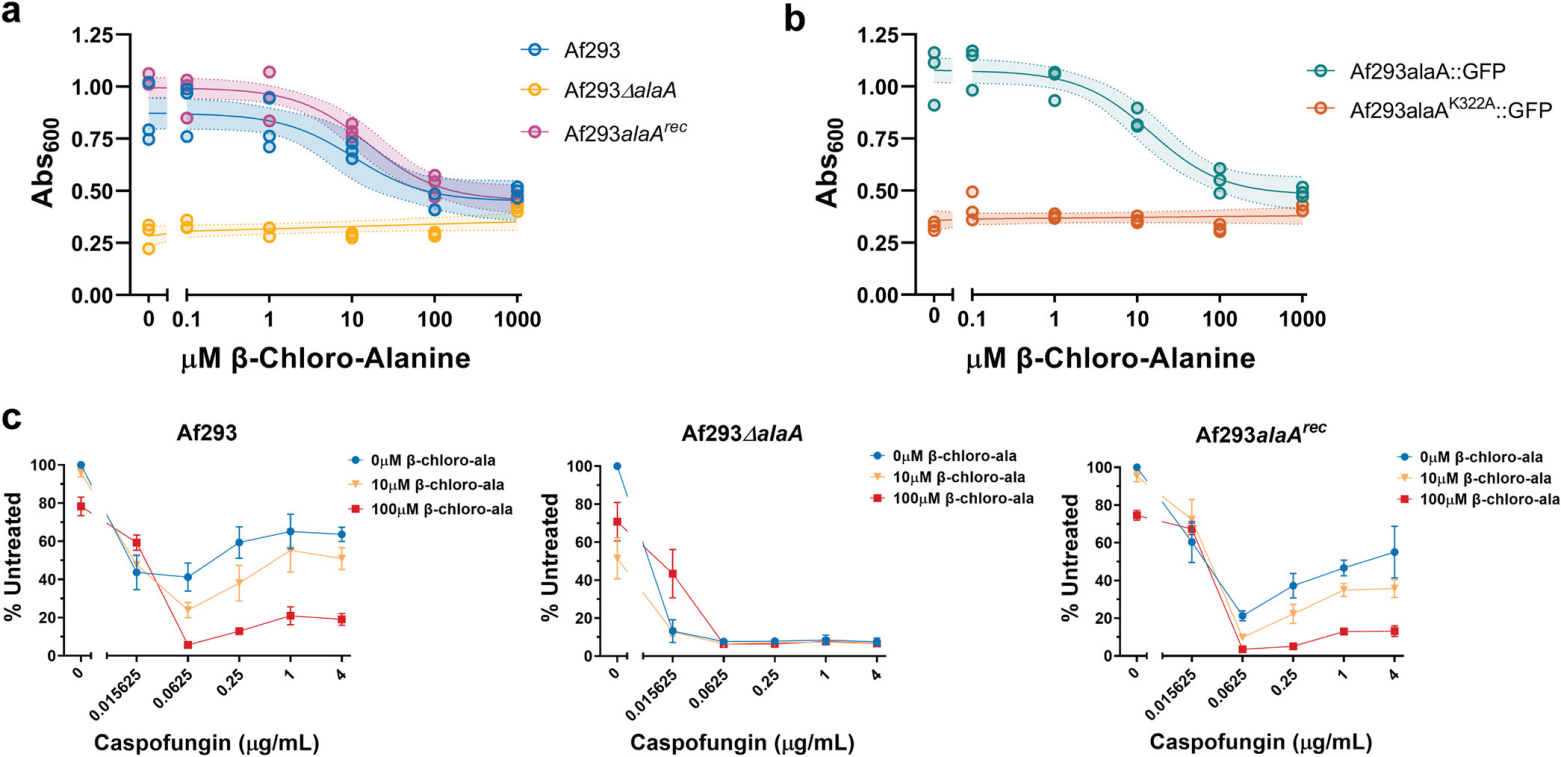
Chemical inhibition of AlaA by β-chloro-l-alanine is sufficient to decrease adherence and increase susceptibility of biofilms to caspofungin. (A and B) Crystal violet adherence assay of 24-h biofilms grown in the presence of increasing concentrations of β-chloro-l-alanine. Each replicate is shown (*n* = 3) along with a nonlinear regression using a dose-response model (line) ± 95% confidence interval (shaded area). (C) Susceptibility of 24-h biofilms of Af293 (left), Af293Δ*alaA* (middle), and Af293*alaA^rec^* (right) strains established in the presence of 0, 10, or 100 μM β-chloro-l-alanine. Biofilms were treated with the indicated concentrations of caspofungin for 3 h, and viability was assessed by XTT assay. Means ± SD are shown (*n* = 3).

Given the potential clinical significance of increasing A. fumigatus biofilm susceptibility to echinocandins, we next tested whether chemical inhibition of AlaA was sufficient to confer similar phenotypes observed in the null or catalytically inactive mutant strains. The chemical β-chloro-l-alanine was previously demonstrated to inhibit mammalian alanine aminotransferases (46, 50, 51), and we tested if β-chloro-l-alanine treatment could recapitulate the adherence and caspofungin phenotypes observed in the *alaA* null strain. Af293, Af293Δ*alaA*, and Af293*alaA^rec^* strains were incubated with 10-fold increasing concentrations of β-chloro-l-alanine from 0.1 μM to 1,000 μM and tested for adherence via crystal violet adherence assay. Increasing concentrations of β-chloro-l-alanine resulted in decreased adherence for Af293 and Af293*alaA^rec^* strains, with EC_50_ values of 10.49 μM and 15.90 μM, respectively (Fig. 6A). At 100 μM β-chloro-l-alanine, adherence of the WT and reconstituted strains was inhibited to slightly above that of the *alaA* null strain. Importantly, adherence of the Af293Δ*alaA* strain was unaltered by any concentration of β-chloro-l-alanine tested, indicating some level of chemical specificity for AlaA (Fig. 6A). Additionally, treatment of the GFP-tagged AlaA and catalytically inactive strains with β-chloro-l-alanine yielded results similar to those for the WT and *alaA* null strains, respectively, suggesting that the compound is acting through the catalytic activity of the AlaA enzyme (Fig. 6B).

To test if β-chloro-l-alanine treatment increases susceptibility of biofilms to echinocandins, biofilms were grown in the presence of 10 μM and 100 μM β-chloro-l-alanine to represent a range of values that encompass both the EC_50_ value determined by the adherence assay results (10 μM) and the concentration that yielded an *alaA* deletion-like phenotype (100 μM) (Fig. 6A). The β-chloro-l-alanine-treated biofilms of Af293, Af293Δ*alaA*, and Af293*alaA^rec^* strains were tested for sensitivity to caspofungin treatment. In the WT and reconstituted strains, efficacy of caspofungin increased as the concentration of β-chloro-l-alanine increased (Fig. 6C). Curiously, even in the *alaA* null strain basal XTT reduction decreased as the concentration of β-chloro-l-alanine increased. However, the *alaA* null strain remained highly susceptible to caspofungin regardless of the concentration of β-chloro-l-alanine (Fig. 6C). Additionally, treatment of CEA10 with β-chloro-l-alanine increased caspofungin susceptibility of biofilms, validating that this is not specific to the Af293 strain background (Fig. S6). Together these data establish the proof of concept that chemical inhibition of AlaA is a possible strategy for increasing susceptibility of A. fumigatus biofilms to echinocandins.

10.1128/mBio.02933-21.6 FIG S6 Chemical inhibition of AlaA by β-chloro-l-alanine increases susceptibility of CEA10 biofilms to caspofungin. Susceptibility of 24-h CEA10 biofilms established in the presence or absence of 100 μM β-chloro-l-alanine to caspofungin treatment. Biofilms were treated with the indicated concentrations of caspofungin for 3 h, and viability was assessed by XTT assay. Means ± SD are shown (*n* = 3). **, *P* < 0.01; ****, *P* < 0.0001 as determined by two-way ANOVA with Tukey’s multiple-comparison test. Download FIG S6, TIF file, 0.5 MB. Copyright © 2022 Kerkaert et al. 2022 Kerkaert et al. https://creativecommons.org/licenses/by/4.0/ This content is distributed under the terms of the Creative Commons Attribution 4.0 International license.

### Altered extracellular matrix is not the primary factor impacting caspofungin susceptibility.

In other fungi, the biofilm extracellular matrix has been determined to be a major factor in reducing antibiotic efficacy against biofilms (52, 53). Therefore, we tested if the increased susceptibility to caspofungin is attributed to the altered GAG composition of the *alaA* null strain. To do this, we utilized a strain lacking the UDP-glucose-4-epimerase required to produce GAG (Af293Δ*uge3*) and a strain lacking the deacetylase required for the maturation of GAG (Af293Δ*agd3*) in combination with β-chloro-l-alanine treatment (19, 23). In an attempt to overcome the inability of Af293Δ*uge3* and Af293Δ*agd3* strains to adhere to abiotic surfaces (19, 23), we performed experiments with these strains on collagen-coated tissue culture plates. Collagen is a mammalian extracellular matrix component abundant in the lung, and we observed that this treatment was sufficient to partially restore adherence of both GAG-deficient strains, indicating the existence of GAG-independent mechanisms of adherence to alternative substrates found in mammalian lungs (Fig. S7A and B). Af293, Af293Δ*uge3*, and Af293Δ*agd3* biofilms were established on collagen-coated plates with or without 100 μM β-chloro-l-alanine and then subsequently treated with caspofungin. All three strains were highly susceptible to caspofungin when AlaA was inhibited by β-chloro-l-alanine (Fig. S7C and D). Untreated Af293Δ*uge3* strain was inhibited by caspofungin treatment to a greater extent than the WT strain. However, this increased susceptibility was far less severe than that observed in β-chloro-l-alanine-treated biofilms and was not observed in the deacetylase-deficient strain (Af293Δ*agd3*) (Fig. S7D). Therefore, GAG contributes to caspofungin resistance to some degree but is not the primary factor responsible for the increased susceptibility when AlaA is chemically inhibited or genetically altered.

10.1128/mBio.02933-21.7 FIG S7 Collagen coating partially rescues adherence of Af293Δ*uge3* and Af293Δ*agd3* strains. (A and B) Crystal violet adherence assay of Af293Δ*uge3* (A) and Af293Δ*agd3* (B) strains in wells of a 96-well plate coated with collagen or PBS (buffer). Each replicate along with the means ± SD are shown (*n* ≥ 3). *, *P* < 0.05; **, *P* < 0.01; ***, *P* < 0.001; ****, *P* < 0.0001 as determined by two-way ANOVA with a Tukey’s multiple-comparison test. (C and D) Susceptibility of 24-h Af293Δ*uge3* (C) and Af293Δ*agd3* (D) biofilms established in the presence or absence of 100μM β-chloro-l-alanine to caspofungin treatment. Biofilms were treated with the indicated concentrations of caspofungin for 3 h and viability was assessed by XTT assay. Means ± SD are shown (*n* = 3). **, *P* < 0.01; ***, *P* < 0.001; ****, *P* < 0.0001 as determined by two-way ANOVA with a Tukey’s multiple-comparison test. The highest *P* values for β-chloro-l-alanine-treated groups versus their respective untreated groups are shown. Download FIG S7, TIF file, 2.5 MB. Copyright © 2022 Kerkaert et al. 2022 Kerkaert et al. https://creativecommons.org/licenses/by/4.0/ This content is distributed under the terms of the Creative Commons Attribution 4.0 International license.

### Mitochondrial perturbation of biofilms is not sufficient to explain echinocandin sensitivity of *alaA*-deficient strains.

Previous studies in several fungal species have revealed links between mitochondrial function and the cell wall integrity pathway that are involved in caspofungin sensitivity (54–57). Thus, we sought to test if the altered function of complex III or complex IV in the *alaA* null strain (Fig. 2F and G) could explain the increased sensitivity of biofilms to caspofungin. To address this, WT Af293 biofilms were grown for 22 h; mitochondrial function was then altered via treatment with several concentrations of the complex III inhibitor antimycin A or the complex IV inhibitor KCN for 2 h. The biofilms were then treated with a combination of caspofungin and the respective mitochondrial inhibitor for 3 h, and viability was assessed by an XTT assay. Treatment of biofilms with either of the inhibitors or caspofungin alone yielded levels of inhibition similar to what was seen in previous assays (Fig. S8A and C). However, when the individual effects of the mitochondrial inhibitors were accounted for, no significant synergistic or additive effects with caspofungin were observed (Fig. S8B and D). Therefore, altered complex III or complex IV activity is not sufficient to explain the increased sensitivity of the *alaA* null strain to caspofungin.

10.1128/mBio.02933-21.8 FIG S8 Altered complex III or complex IV function is not sufficient to explain increased caspofungin sensitivity. WT Af293 biofilms were grown for 22 h, followed by a 2-h treatment with the indicated concentrations of the complex III inhibitor antimycin A or the complex IV inhibitor KCN to disturb mitochondrial function. Caspofungin and fresh antimycin A or KCN were then applied at the indicated concentrations to each biofilm for three hours. Viability was assessed by XTT assay. (A and C) To observe the individual effects of each drug, data were normalized to the untreated control (0 μg/mL mitochondrial inhibitor, 0 μg/mL caspofungin). (B and D) To test for synergy, data were normalized to the no caspofungin control for each respective concentration of the mitochondrial inhibitor. Means ± SD are shown (*n* = 4). Download FIG S8, TIF file, 2.3 MB. Copyright © 2022 Kerkaert et al. 2022 Kerkaert et al. https://creativecommons.org/licenses/by/4.0/ This content is distributed under the terms of the Creative Commons Attribution 4.0 International license.

### *alaA* is required for echinocandin resistance *in vivo*.

**FIG 7:**
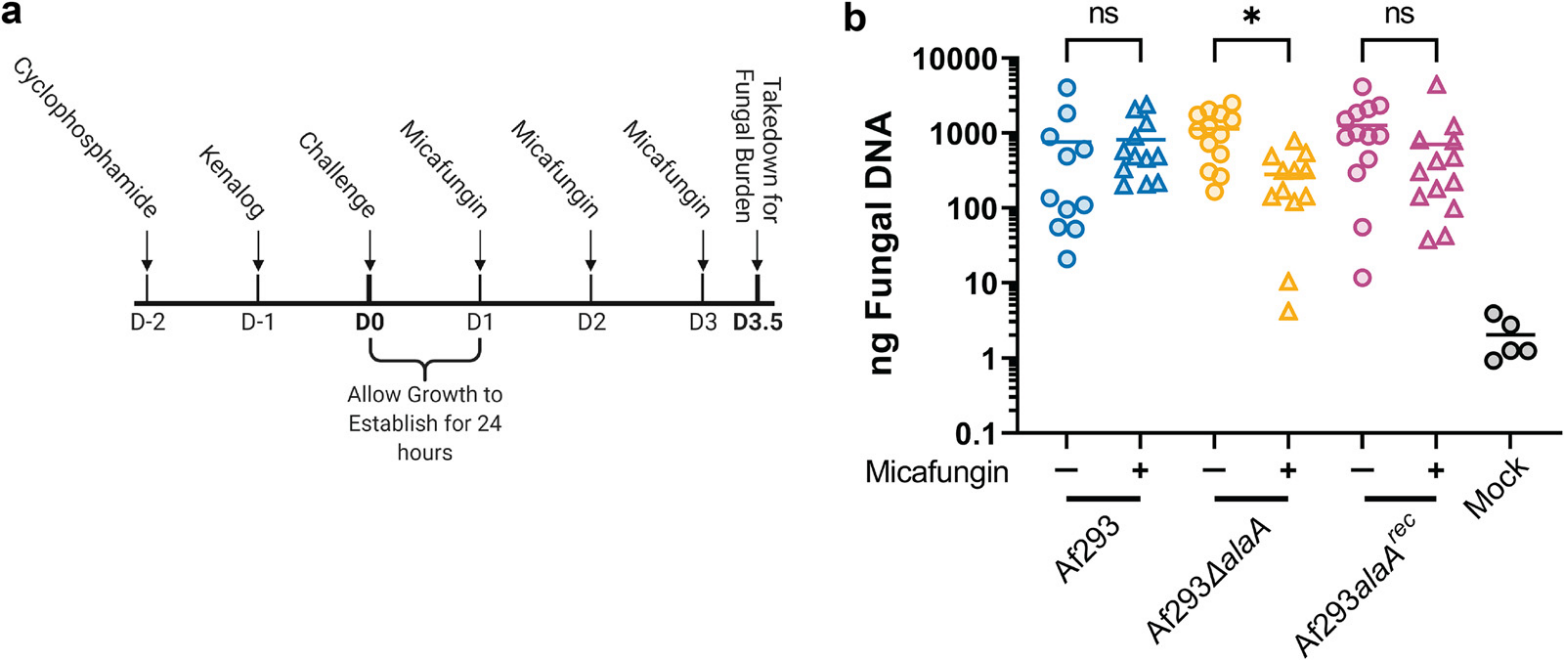
*alaA* is required for echinocandin resistance *in vivo*. (A) Experimental outline for determining *in vivo* echinocandin resistance using a chemotherapy model of invasive aspergillosis. Outbred CD1 mice were immunosuppressed with 150 mg/kg cyclophosphamide 48 h prior to fungal challenge and 40 mg/kg triamcinolone 24 h prior to fungal challenge. Mice were challenged with A. fumigatus or PBS (mock) at day 0 (D0), and infection was allowed to establish for 24 h. Mice were treated with either 1 mg/kg micafungin or 0.9% NaCl every 24 h from D1 to D3; 12 h after the final micafungin treatment, mice were sacrificed for fungal burden determination by qPCR. (B) qPCR quantification of total nanograms of fungal DNA in lungs of mice challenged with the indicated A. fumigatus strains and left untreated or treated with 1 mg/kg micafungin according to the design in panel A. Each data point and the means are shown (*n* ≥ 12 for each experimental group and *n* = 5 for mock-infected mice across two independent experiments). *, *P* < 0.05; ns, not significant as determined by Kruskal-Wallis with a Dunn’s multiple-comparison test.

Finally, we sought to determine if *alaA* plays a role in echinocandin resistance *in vivo* within lung infection microenvironments. To address this question, we utilized a chemotherapy murine model of invasive pulmonary aspergillosis (IPA). Outbred CD1 mice were immunosuppressed with cyclophosphamide and triamcinolone and then challenged with conidia of Af293, Af293Δ*alaA*, or Af293*alaA^rec^* strains. The infection was allowed to establish for 24 h, followed by three treatments with either 0.9% NaCl or 1 mg/kg of body weight micafungin every 24 h (Fig. 7A); 12 h after the final micafungin treatment, relative fungal burden was determined by quantitative PCR (qPCR) quantification of A. fumigatus 18S rDNA. The dose of micafungin used had no significant impact on fungal burden in mice inoculated with the WT or reconstituted strains. Moreover, loss of *alaA* at the time point examined did not significantly impact fungal burden levels in the untreated groups (Fig. 7B). In contrast, there was a 4-fold reduction in fungal burden in mice inoculated with Af293Δ*alaA* strain and treated with micafungin compared to untreated mice (Fig. 7B). Thus, loss of *alaA in vivo* significantly increases the susceptibility of A. fumigatus to subeffective concentrations of the echinocandin micafungin.

## DISCUSSION

In this study, we describe the first gene in A. fumigatus that has been found to confer biofilm-specific resistance to an antifungal drug. Genetic or chemical disruption of AlaA’s catalytic activity was sufficient to greatly increase sensitivity of biofilms to echinocandins, despite *alaA* being dispensable for resistance when the drug was applied to conidia. Our results highlight the concept that an organism will respond differently to a drug/stress depending on the growth state it is in and thereby may require different mechanisms for resistance to said drug/stress. Clinical treatment of A. fumigatus is largely in the context of an already established infection where the fungus exists in a biofilm state. Despite this, the vast majority of antifungal drug studies and discoveries have been based on treatment of the conidial state of A. fumigatus or the planktonic state of other fungi that clinically present as a biofilm.

Given the results presented in this work as well as others, we have demonstrated that antifungal treatment of biofilms is far less effective than treatment of conidia (27, 28). Mechanisms to increase efficacy of antifungal drugs in the context of A. fumigatus biofilms are of great clinical need and may even bridge the discrepancies between relatively low, but rising, rates of antifungal resistance *in vitro* and the relatively high rates of clinical treatment failure (10–13). Both genetic and chemical disruption of AlaA resulted in increased susceptibility of biofilms to echinocandin treatment, suggesting inhibition of this enzyme is a treatment option for clinically enhancing the efficacy of echinocandins. While alanine aminotransferase inhibitors have been developed (46, 50, 51), these molecules were discovered using mammalian alanine aminotransferases and would impact both the host and the pathogen (AlaA has 45.77% and 45.19% amino acid sequence identity with human GPT1 and GPT2, respectively [see Fig. S2B and C in the supplemental material]). Thus, the chemical inhibition performed in this study serves as a proof of principle for targeting AlaA activity in a combination therapy approach. However, these molecules could provide chemical building blocks for development of more fungus-specific and/or potent alanine aminotransferase inhibitors. β-Chloro-l-alanine has been utilized in a murine cancer model where inhibition of murine alanine aminotransferase was found to decrease the Warburg effect and increase mitochondrial activity of tumor cells (46). While this is promising for future studies *in vivo*, further safety and pharmacological studies are needed before these inhibitors are utilized in the context of an infection model.

We originally found *alaA* through investigation of data sets associated with low-oxygen adaptation. AlaA catalyzes the interconversion of pyruvate and alanine without direct involvement of reducing potentials or any high-energy molecules, such as ATP. However, the reduction of nitrate to alanine would consume five reducing potentials, and this pathway is suggested to be important for a variety of systems in the adaptation to low oxygen (35, 36, 58–60). It is possible that in these systems alanine serves as a nitrogen sink to prevent toxic ammonium accumulation during the conversion of nitrate to ammonium. Therefore, we had originally hypothesized that AlaA function was a critical means of recycling reducing potentials during low-oxygen growth and were surprised to find that AlaA plays a significant role in polysaccharide regulation and biofilm formation despite the minimal impact on growth in a low-oxygen environment. This result could be due to a high redundancy in the number of mechanisms encoded by the fungus to balance reducing potentials, or it could suggest that alanine metabolism has a more specific role in adaptation to natural oxygen gradients formed by respiration and/or adaptation to stochastic fluctuations in environmental oxygen that naturally occur during filamentous fungal biofilm growth.

AlaA is one of two enzymes encoded by the A. fumigatus genome capable of generating alanine, the other being a putative alanine-glyoxylate aminotransferase (Afu1g09470). It was also recently demonstrated that the GatA (Afu5g06680) homolog in S. cerevisiae, Uga1, is capable of generating alanine using GABA as the amino-group donor, making GatA a potential third enzyme capable of generating alanine (42). Despite *alaA* being critical for numerous physiological aspects of the A. fumigatus biofilm, these other two pathways appear to be sufficient for maintaining basal levels of alanine for protein production, as genetic disruption of *alaA* did not yield alanine auxotrophy. This, combined with the severe growth defects on alanine as a sole carbon or nitrogen source, point to AlaA as having a primarily catabolic role. The involvement of an alanine aminotransferase in polysaccharide regulation is particularly perplexing when one considers that none of the components of the reaction (alanine, pyruvate, glutamate, and α-ketoglutarate) have any obvious or direct role in any known biochemical pathways that generate A. fumigatus cell wall or extracellular matrix components. While the exact mechanism by which *alaA* regulates these processes remains an active area of investigation, the findings in this study significantly narrow down the potential hypotheses and suggest the mechanism of regulation to be novel with regard to antifungal drug resistance.

The *alaA* homolog in S. cerevisiae, *alt1* (49.7% amino acid identity [Fig. S2C]), has also been described to have broad impacts on cell physiology, including a high-throughput screen yielding a moderate increase in sensitivity to caspofungin (42, 43, 61). While the caspofungin phenotype has not been specifically investigated beyond the screen, many of the phenotypes conferred by loss of *alt1* have been demonstrated to function through a catalytic-independent, or moonlighting, function (42, 43). In contrast, here we described that the catalytic activity of the AlaA protein is essential for polysaccharide regulation rather than the presence or absence of the protein itself, as would be the case for a moonlighting function. Therefore, while alanine aminotransferases in both A. fumigatus and S. cerevisiae have broad impacts on physiology, the mechanisms by which they confer these broad impacts are distinctly different.

In addition to ruling out a moonlighting function, we rule out two known mechanisms that alter antifungal susceptibility in other systems, matrix-mediated resistance to antifungals and mitochondrial disruption-mediated alterations to echinocandin resistance. Therefore, some aspect of the catalyzed reaction is involved, suggesting several possibilities, including that the reaction is a critical regulator of metabolic flux, whereby disruption of the reaction feeds back to alterations in polysaccharide precursor levels, that a molecule impacted by the reaction is an allosteric regulator of several polysaccharide-modifying enzymes (e.g., Agd3), or that a molecule impacted by the reaction is playing a role as a signaling molecule to yield broad physiological impacts on the cell/biofilm. With regard to the latter two hypotheses, alanine has been found to act as a signaling molecule for spore germination in Bacillus subtilis via the GerA receptor proteins (62–64). Another factor of great interest is the biofilm-specific nature of this mechanism. Further investigation into these phenomena and their underlying mechanism could yield significant insight into the interplay between metabolism, biofilm formation, and antifungal drug resistance with the potential to inform development of novel biofilm targeted antifungal therapeutics.

## MATERIALS AND METHODS

### Strains and growth conditions.

Mutant strains were made in the Aspergillus fumigatus Af293 (68) and CEA10/CBS144.89 (69) (referred to as CEA10 throughout the manuscript) strains; therefore, Af293 and CEA10/CBS144.89 were used as the wild-type (WT) strains as appropriate for each experiment. Strains were stored as conidia in 25% glycerol at −80°C and maintained on 1% glucose minimal medium [GMM; 6 g/liter NaNO_3_, 0.52 g/liter KCl, 0.52 g/liter MgSO_4_·7H_2_O, 1.52 g/liter KH_2_PO_4_ monobasic, 2.2 mg/liter ZnSO_4_·7H_2_O, 1.1 mg/liter H_3_BO_3_, 0.5 mg/liter MnCl_2_·4H_2_O, 0.5 mg/liter FeSO_4_·7H_2_O, 0.16 mg/liter CoCl_2_·5H_2_O, 0.16 mg/liter CuSO_4_·5H_2_O, 0.11 mg/liter (NH_4_)_6_Mo_7_O_24_·4H_2_O, 5 mg/liter Na_4_EDTA, 1% glucose; pH 6.5]. Solid medium was prepared by addition of 1.5% agar. All experiments were performed with GMM unless explicitly stated otherwise. For experiments where alanine was the sole carbon or nitrogen source, glucose or NaNO_3_ was replaced with alanine at an equimolar quantity of carbon or nitrogen atoms, respectively. For all experiments, A. fumigatus was grown on solid GMM at 37°C 5% CO_2_ for 3 days to produce conidia. Conidia were collected using 0.01% Tween 80, counted using a hemacytometer, and diluted in either 0.01% Tween 80 or medium to the final concentration used in each assay.

### Strain construction.

*alaA* null mutants were generated by replacing the *alaA* open reading frame (AFUB_073730/Afu6g07770) with the dominant selection marker *ptrA* in both the Af293 and CEA10 backgrounds. The replacement construct was generated using overlap PCR to fuse ∼1 kb upstream and ∼1 kb downstream of the open reading frame of *alaA* to the *ptrA* marker. The resulting construct was transformed into protoplasts of each strain, and mutants were selected for on osmotically stabilized minimal medium (GMM plus 1.2 M sorbitol) containing 100 μg/liter pyrithiamine hydrobromide (Sigma). Reconstitution of the *alaA* gene was performed by PCR amplification of the *alaA* locus for each strain from ∼1.1 kb upstream of the start codon to ∼700 bp downstream of the stop codon using primers containing PacI and AscI digestion sites. The resulting PCR products were digested with PacI and AscI restriction enzymes and individually ligated into a plasmid containing the hygromycin resistance marker *hygR*. The resulting plasmids were ectopically transformed into protoplasts derived from the *alaA* null strain for each plasmid’s respective background. Reconstituted mutants were selected for on osmotically stabilized minimal medium containing both 175 μg/mL hygromycin B (VWR) and 100 μg/liter pyrithiamine to ensure the mutated locus remained intact. GFP-tagged alleles of WT *alaA* and catalytically inactive *alaA^K322A^* were generated at the *alaA* native locus in the Af293 background using the *ptrA* marker. The WT allele was generated using overlap PCR to fuse ∼1 kb upstream of the stop codon of *alaA*, excluding the stop codon, to a fragment containing an in-frame *gfp* linked to a *trpC* terminator from A. nidulans and the *ptrA* marker, along with the same ∼1 kb downstream of the stop codon that was used in the deletion construct. The catalytically inactive mutation was generated using nested PCR from the mutation site to immediately before the stop codon to modify the AAG lysine codon to a GCC alanine codon. This fragment was then fused with 500 bp upstream of the point mutation, along with the in-frame gfp-trpC_terminator_ *ptrA* fragment, and ∼1 kb downstream of the *alaA* stop codon. The two alleles were transformed into Af293 protoplasts, and mutants were selected using pyrithiamine. Sanger sequencing was used to confirm each allele.

Protoplasts were generated using lysing enzyme from *Trichoderma harzianum* (Sigma) and transformed as previously described (32). Protoplasts were plated on sorbitol stabilized minimal medium (GMM plus 1.2 M sorbitol) containing pyrithiamine. For hygromycin selection, protoplasts were allowed to recover without hygromycin selection until germ tubes were visible by inverted microscope (overnight at 37°C), at which point 0.6% agar medium containing hygromycin was added to a final concentration of 175 μg/mL. All strains were single spored and checked for correct integration, or the presence of construct in the case of the ectopic reconstituted strains, via PCR and Southern blotting. Additionally, the basal expression of *alaA* in the reconstituted strains was checked by reverse transcription-qPCR (RT-qPCR) on RNA extracted from 24-h biofilms using the *alaA* null mutants as negative controls (Fig. S9). All strains generated in this study are available upon request from the corresponding author.

10.1128/mBio.02933-21.9 FIG S9 RT-qPCR of *alaA* expression in *alaA* deletion and reconstituted strains. (A and B) RNA was harvested from 24-h biofilms and *alaA* expression was determined by RT-qPCR (*n* = 3). Each replicate along with the median are shown. *, *P* < 0.05; **, *P* < 0.01; ***, *P* < 0.001; ns, not significant as determined by one-way ANOVA with a Tukey’s multiple-comparison test. Download FIG S9, TIF file, 0.9 MB. Copyright © 2022 Kerkaert et al. 2022 Kerkaert et al. https://creativecommons.org/licenses/by/4.0/ This content is distributed under the terms of the Creative Commons Attribution 4.0 International license.

### Growth and conidial viability assays.

For assays of growth on alanine as a carbon or nitrogen source, an equal molarity of carbon or nitrogen atoms was added for each indicated molecule to our base minimal medium lacking NaNO_3_ and glucose. Agar plates were inoculated with 10^3^ conidia and incubated for 72 h at 37°C, 5% CO_2_ in either ambient oxygen or in a chamber that maintained oxygen at a concentration of 0.2% (InvivO_2_ 400 Workstation; Ruskinn Baker). Biofilm biomass cultures were inoculated with 20 mL of 10^5^ conidia/mL in GMM and grown in petri plates for 24 h at 37°C, 5% CO_2_. Supernatants and air-liquid interface growth were removed, and biofilms were harvested using a cell scraper. Biomass was washed 2× with double-distilled water with centrifuging at 5,000 rpm for 10 min to spin down biomass, frozen at −80°C, and lyophilized, and dry weight was measured. For liquid kinetic growth assays of biofilms, 200 μL of 10^5^ conidia/mL in GMM was inoculated in six technical replicates per strain in a 96-well plate. Plates were incubated statically in a plate reader at 37°C with *A*_405_ readings every 15 min over the first 24 h of growth. Conidia viability was assessed by plating ∼100 conidia in 5 mL of 0.6% top-agar GMM onto a petri plate of GMM and counting CFU after incubation at 37°C 5% CO_2_.

### Crystal violet adherence assay.

U-bottomed 96-well plates were inoculated with 10^5^ conidia/mL in GMM and incubated statically for the indicated time at 37°C, 5% CO_2_ to allow biofilms to form. To remove nonadherent cells, medium was removed and biofilms were washed twice with water via immersion followed by banging the plate onto a stack of paper towels. Adherent biomass was stained with 0.1% (wt/vol) crystal violet for 10 min, and biofilms were washed twice with water to remove excess crystal violet. Crystal violet was then dissolved in 100% ethanol, supernatants were transferred to a flat-bottomed plate, and absorbance at 600 nm was quantified. Dose-response assays testing the impact of β-chloro-l-alanine on adherence were fit with a nonlinear regression based on a dose-response model (GraphPad Prism 9) to calculate 50% effective concentration values. For assays testing the impact of collagen coating, 50 μL of collagen coating solution (Sigma) was applied to half the wells of a 96-well U-bottom plate overnight at room temperature. The solution was removed, and wells were washed one time with phosphate-buffered saline (PBS) prior to inoculation. All crystal violet adherence assays were performed with 3 to 6 technical replicates, and data presented represent at least three biological replicates.

### Oxygen quantification.

Oxygen was quantified as previously described (27) using a Unixense oxygen measuring system 1-CH (Unisense OXY METER) equipped with a micromanipulator (Unisense MM33), motorized micromanipulator stage (Unisense MMS), motor controller (Unisense MC-232), and a 25-μm Clark-type/amperometric oxygen sensor (Unisense OX-25). The SensorTrace Suite Software v3.1.151 (Unisense) was utilized to obtain and analyze the data. Falcon 35-mm petri dishes (Fisher) were coated with 2 mL of 0.6% agar GMM to protect the microelectrode from breaking when performing deep profiling into the biofilms; 3 mL of 10^5^ conidia/mL in GMM was inoculated into the plates and incubated for 24 h at 37°C, 5% CO_2_. The meniscus of the culture was ∼3 mm above the surface of the agar pad, and oxygen was measured at the center of each culture in 200-μm steps, with technical duplicates at each step, from the air-liquid interface to 2,800 μm into the culture. Oxygen quantification was performed immediately upon removal of the culture from the incubator. At least seven independent biofilms were measured for each strain across two experiments along with three medium-only cultures that lacked fungus.

### Fluorescent microscopy.

Fluorescent confocal microscopy was performed on an Andor W1 spinning disk confocal microscope with a Nikon Eclipse Ti inverted microscope stand.

### (i) AlaA localization studies.

Af293*alaA-GFP*, Af293*alaA^K322A^-GFP*, and Af293 strains were cultured in GMM on MatTek dishes at 37°C in GMM until germlings were visible on an inverted light microscope, ∼9 h for Af293*alaA-GFP* and Af293 strains, and ∼10 h for Af293*alaA^K322A^-GFP* strain. Medium was removed and replaced with fresh GMM containing 100 nm MitoTracker Deep Red FM (ThermoFisher). Cultures were incubated for 30 min at 37°C to allow mitochondrial staining. Images were acquired with a 60× oil immersion objective at 488 nm (GFP) and 637 nm (MitoTracker) on the Andor W1 spinning disk confocal microscope. Images were deconvolved, and max intensity z-projections were generated using the Nikon NIS-Elements AR software. Experiment was also performed with WT Af293 as a negative control for autofluorescence (see Fig. S2D in the supplemental material). At least 10 images for each strain were taken across four replicate cultures.

### (ii) Fungal biofilm imaging and quantification.

Biofilms were grown in 2 mL of GMM at 10^5^ conidia/mL in 35-mm glass bottom MatTek dishes for the indicated time at 37°C, 5% CO_2_. At the indicated time, 1 mL of medium was removed and 500 μL of 30 μg/mL FITC-SBA (Vector Laboratories) was added to each culture. Cultures were incubated at room temperature for 1 h to allow staining. Biofilms were then fixed via addition of 500 μL 4% paraformaldehyde in PBS and counterstained with 200 μL of 275 μg/mL calcofluor white (Fluorescent Brightener-28 [Sigma]). Images of the first ∼300 μm of the biofilms were acquired on the Andor W1 spinning disk confocal microscope with a 20× multi-immersion objective (Nikon) at 405 nm (calcofluor white) and 488 nm (FITC-SBA).

For quantification and analysis of biofilms, the BiofilmQ framework was used. A detailed explanation of BiofilmQ can be found in a previous publication (47). Briefly, both biomass (calcofluor white) and matrix (FITC-SBA) were thresholded and then segmented into discrete objects with 20-voxel cubes for further analysis. Total biovolume measures were achieved by taking the summed volume of the segmented calcofluor white signal for each biofilm. Total matrix intensity was determined by summing the intensity signal of FITC-SBA in each cube of segmented matrix signal across the entire image. We measured hyphal associated matrix by taking the sum of FITC-SBA intensity overlapping in each cube of segmented biomass. Representative images were rendered using the VTK output feature of BiofilmQ. These files could then be rendered in ParaView (65) using Ospray ray tracing. Matrix intensity per individual cube as determined in BiofilmQ was then mapped onto the segmented matrix images.

### (iii) Cell wall staining and quantification.

Strains were grown in GMM in the center of MatTek dishes until germlings were visible by an inverted light microscope, ∼9 h for Af293 and Af293*alaA^rec^* strains and ∼10 h for Af293Δ*alaA* strain. Supernatants were removed and cells were washed with PBS. For calcofluor white staining, germlings were fixed with 4% paraformaldehyde for 15 min, washed with PBS, and stained with 25 μg/mL calcofluor white (Fluorescent Brightener 28; Sigma) in PBS for 15 min. Calcofluor white was removed, and germlings were washed with PBS and maintained in 2 mL PBS at room temperature until imaging. For FITC-WGA staining, germlings were stained with 5 μg/mL FITC-WGA in GMM for 30 min at room temperature. Germlings were washed with PBS and fixed with 4% paraformaldehyde for 15 min. WGA-stained germlings were then washed with PBS and maintained in 2 mL PBS at room temperature until imaging. Finally, Dectin-1-stained germlings were fixed with 4% paraformaldehyde for 15 min and washed with PBS. Blocking solution (RPMI plus 10% fetal calf serum plus 0.025% Tween 20) was applied for 1 h at room temperature. Blocking solution was removed and 5 μg/mL of Dectin-1-Fc in blocking solution was applied for 1 h at room temperature. Germlings were washed with PBS, and the secondary antibody Alexa Fluor 488 anti-human IgG (ThermoFisher) was added at a 1/300 dilution in PBS for 1 h at room temperature. Germlings were washed a final time with PBS, and cells were maintained in 2 mL PBS until imaging. All staining took place in the dark, and extreme care was taken to not disrupt the Af293Δ*alaA* germlings.

All germlings were imaged on the Andor W1 spinning disk confocal microscope with a 60× oil immersion objective using 405 nm for calcofluor white and 488 nm for FITC-WGA and Dectin-1-Fc. Cell wall staining was quantified using Fiji (ImageJ). Z-stacks were assembled using a sum-intensity Z-projection. Regions of interest (ROIs) were drawn around each individual germling within a given image along with a region lacking any germlings to account for background fluorescence. Within each ROI the area, sum intensity, and mean intensity were quantified. To obtain corrected mean intensity measurements, the mean background intensity was multiplied by the area of the ROI to calculate total background contribution. The total background contribution was subtracted from the ROI’s sum intensity, and this value was divided by the area of the ROI yielding the final corrected mean fluorescence intensity. Each cell wall stain was performed in triplicate cultures, and at least three fields of view were obtained for each culture. For FITC-WGA the staining pattern was almost entirely absent from the germ tube, and enough natural size heterogeneity was found both within and between cultures to act as a confounding variable. Thus, for FITC-WGA ROIs were drawn around each conidial body where the staining was present rather than the entire germling.

### Extracellular matrix monosaccharide analysis and ELLA. (i) ELLA.

A volume of 100 μL of 10^5^ conidia per mL in GMM was inoculated into wells of 96-well plates and incubated for 24 h. Culture supernatants were then transferred to a 384-well plate Immulon 4HBX with or without 500 pM recombinant Agd3. After a 1-h incubation period, wells were washed three times with 1× Tris-buffered saline–0.05% Tween 20 (TBS-T). A preincubated solution of 30 nM soybean agglutinin lectin coupled to biotin and 1/700 avidin-horseradish peroxidase in TBS-T was added to the wells and incubated for 1 h. After 3 TBS-T washes, detection was performed using Ultrasensitive TMB read at 450 nm. Normalization of the values was performed by comparing the absorbance reads to the absorbance of Af293.

### (ii) Extracellular matrix monosaccharide composition by gas chromatography coupled to mass spectrometry.

A volume of 100 mL of GMM was inoculated with 10^4^ conidia per mL and incubated for 3 days at 37°C at 200 rpm. Culture supernatants were filtered by Miracloth prior to being dialyzed for 3 days against Milli-Q water and lyophilized. About 0.5 mg of dried material was then derivatized into trimethylsilyl derivatives. Samples were hydrolyzed with either 2 M trifluoroacetic acid for 2 h at 110°C or 6 M hydrochloric acid for 4 h at 100°C. Monosaccharides were then converted in methyl glycosides by heating in 1 M methanol-HCl (Sigma-Aldrich) for 16 h at 80°C. Samples were dried and washed twice with methanol prior to re-N-acetylating hexosamine residues. Re-N-acetylation was performed by incubation with a mix of methanol, pyridine, acetic anhydride (10:2:3) for 1 h at room temperature. Samples were then treated with hexamethyldisilazane-trimethylchlorosilane-pyridine solution (3:1:9; ThermoFisher) for 20 min at 110°C. The resulting TMS methyl glycosides were dried, resuspended in 1 mL of cyclohexane, and injected in an Agilent 7890B GC-5977A MSD. Identification and quantification of the monosaccharides was performed using a mix of monosaccharide calibrants injected at different concentrations as a reference. Quantification was finally normalized to an equivalent of 1 mg of material before comparison between groups.

### RNA extraction and RT-qPCR.

RNA was extracted from 24-h biofilm cultures in a 6-well plate. Supernatant was removed and 500 μL of TRIsure (Bioline Reagents) was immediately applied to the biofilms. Biofilm suspensions were centrifuged, and supernatant was removed. Biomass was resuspended in 200 μL TRIsure, flash frozen in liquid nitrogen, and subjected to bead beating with 2.3-mm beads. Homogenate was brought to a final volume of 1 mL with TRIsure and bead beaten a second time, and RNA was extracted by following the manufacturer’s protocol. A sample of 5 μg of RNA was DNase treated with the TURBO DNA-free kit (Invitrogen) according to the manufacturer’s protocol. A sample of 500 ng of DNase-treated RNA was run on an agarose gel to ensure RNA integrity, and 500 ng of DNase-treated RNA was used for cDNA synthesis as previously described (66). The RT-qPCR data were collected on a CFX Connect real-time PCR detection system (Bio-Rad) with CFX Maestro Software (Bio-Rad). Gene expression was normalized to *tefA* expression for all experiments. The following primer sets were used to quantify expression of each gene: *uge3*, CGACCCAGAATGGACTAT and ACGACGACAGGAAGTAAG; *agd3*, GTGGGTTGAGACGATTG and AAGGAAGTTCTCGGACAT; *alaA*, GGTGATCGGTCAGTGCCTGG and GGCTTCGTACAGGGCGAGG; *tefA*, GTGACTCCAAGAACGATCCC and AGAACTTGCAAGCAATGTGG.

### Antifungal drug and mitochondrial inhibitor susceptibility. (i) Biofilm assays.

To test the susceptibility of biofilms to inhibition by mitochondrial inhibitors, calcofluor white, and caspofungin, 500 μL of 10^5^ conidia per mL in GMM was inoculated into wells of 24-well plates, and biofilms were grown statically for 24 h at 37°C 5% CO_2_. Any air-liquid interface growth was removed using a sterile pipette tip, the supernatant was removed from each well, and fresh medium containing the indicated concentration of rotenone, antimycin A, KCN, calcofluor white, or caspofungin was added to the biofilm. Biofilms were incubated for a further 3 h at 37°C 5% CO_2_ and washed with PBS, and 300 μL of XTT solution was added to each well (0.5 mg/mL XTT [VWR] with 25 μM menadione in PBS). XTT solution was incubated for 1 h at 37°C to allow reduction of the dye; 150 μL of supernatant was transferred to a flat-bottomed 96-well plate, and the absorbance at 450 nm was read on a plate reader. *A*_450 _values of the treated samples were compared to untreated biofilms to calculate the relative metabolic activity as the percentage of untreated control for each strain. All XTT assays were performed on at least three biological replicates.

To test if mitochondrial perturbation of biofilms impacted caspofungin sensitivity, the same assay was performed with the following modifications. Biofilms were grown for 22 h, and then fresh medium containing the indicated concentration of antimycin A or KCN was added. Biofilms were incubated for 2 h to allow disturbance of mitochondrial function, and then medium was replaced with fresh medium containing both the indicated concentration of antimycin A or KCN and the indicated concentration of caspofungin. Biofilms were further incubated for 3 h, followed by assessment of viability by XTT assay.

For the adenylate kinase release assay, biofilms were grown and treated in the same manner as that described above. After 3 h of echinocandin treatment, supernatants were collected and allowed to cool to room temperature. XTT assay was performed on the biofilms according to the protocol described above for matched XTT data. Relative adenylate kinase levels were measured on 40 μL of supernatants via the ToxiLight Non-Destructive Cytotoxicity BioAssay kit (Lonza) according to the manufacturer’s instructions. Chemiluminescence was measured on a Synergy Neo2 multimode plate reader (BioTek). The experiment was performed on four independent biological replicates.

### (ii) Conidia assays.

Minimal effective concentration (MEC) assays were performed by inoculating 100 μL of 2 × 10^5^ conidia/mL in GMM into 96-well flat-bottomed plates containing 100 μL serial 2-fold dilutions of caspofungin in GMM from 8 μg/mL to 0.015625 μg/mL, along with a no drug control. Cultures were grown statically for 24 h and viewed under an inverted light microscope for the concentration at which gross morphological changes characteristic of caspofungin treatment became visible. This concentration was deemed the MEC. Radial growth assays were performed by inoculating GMM agar plates containing the indicated quantity of caspofungin with 10^3^ conidia in 2 μL 0.01% Tween 80 and incubating at 37°C, 5% CO_2_ for 72 h. Images are representative of four biological replicates.

### Murine fungal burden assay.

All mice were housed in autoclaved cages at 3 to 4 mice per cage and provided food and autoclaved water *ad libitum*. Female outbred CD-1 mice (Charles River Laboratory), 20 to 24 g, were immune suppressed with 150 mg/kg cyclophosphamide (Ingenus Pharmaceuticals, LLC) intraperitoneally 48 h prior to inoculation and 40 mg/kg triamcinolone acetonide (Kenalog-10; Bristol-Myers Squibb) subcutaneously 24 h prior to fungal challenge. Mice were administered 10^6^ conidia in 30 μL PBS intranasally under isoflurane anesthesia. Mock mice were administered 30 μL sterile PBS. Micafungin treated mice were administered 1 mg/kg micafungin (Mycamine; Astellas Pharma) intraperitoneally at 24, 48, and 72 h postfungal inoculation. Untreated mice were administered 100 μL 0.9% saline (vehicle control) intraperitoneally at 24, 48, and 72 h postfungal inoculation. Mice were sacrificed at 84 h postfungal inoculation, and lungs were harvested for fungal burden.

Lungs were divided between two 2-mL screw cap tubes and physically chopped using dissecting scissors, flash frozen in liquid nitrogen, and lyophilized for 48 h. The freeze-dried lungs were then bead beaten with 2.3-mm Zirconia beads, and DNA was extracted using the E.Z.N.A. fungal DNA minikit (Omega Bio-tek) with the following modifications. Bead-beaten lungs were resuspended in 600 μL FG1 buffer, bead beaten a second time, and incubated at 65°C for 1 h. Samples were centrifuged, and supernatants from the split lung samples were combined in a new tube. The protocol was continued with 200 μL of the combined supernatant according to the manufacturer’s instructions with two elution steps using 100 μL molecular-grade water heated to 65°C. qPCR quantification of fungal DNA was performed as previously described (67). The fungal burden experiment was performed two times with *n* ≥ 6 in each experimental group per experiment and *n* = 5 mock across the two experiments. Four mice across the two experiments, including one in the Af293Δ*alaA* strain-treated group, were censored for either unsuccessful infection or fungal DNA extraction based on the criteria of having less fungal DNA than the highest mock control value.

### Statistics and reproducibility.

All statistical analyses were performed in GraphPad Prism 9. Unless otherwise noted, all experiments were performed with a minimum of three biologically independent samples.

10.1128/mBio.02933-21.10 TABLE S1 Fungal strains used in this study. Download Table S1, DOCX file, 0.02 MB. Copyright © 2022 Kerkaert et al. 2022 Kerkaert et al. https://creativecommons.org/licenses/by/4.0/ This content is distributed under the terms of the Creative Commons Attribution 4.0 International license.
