# Supplementary figures and images for: An Alanine Aminotransferase Is Required for Biofilm-Specific Resistance of Aspergillus fumigatus to Echinocandin Treatment
Source: mBio. 2022 Mar 7;13(2):e02933-21. doi: 10.1128/mbio.02933-21 (PMC9040767; doi:10.1128/mbio.02933-21)

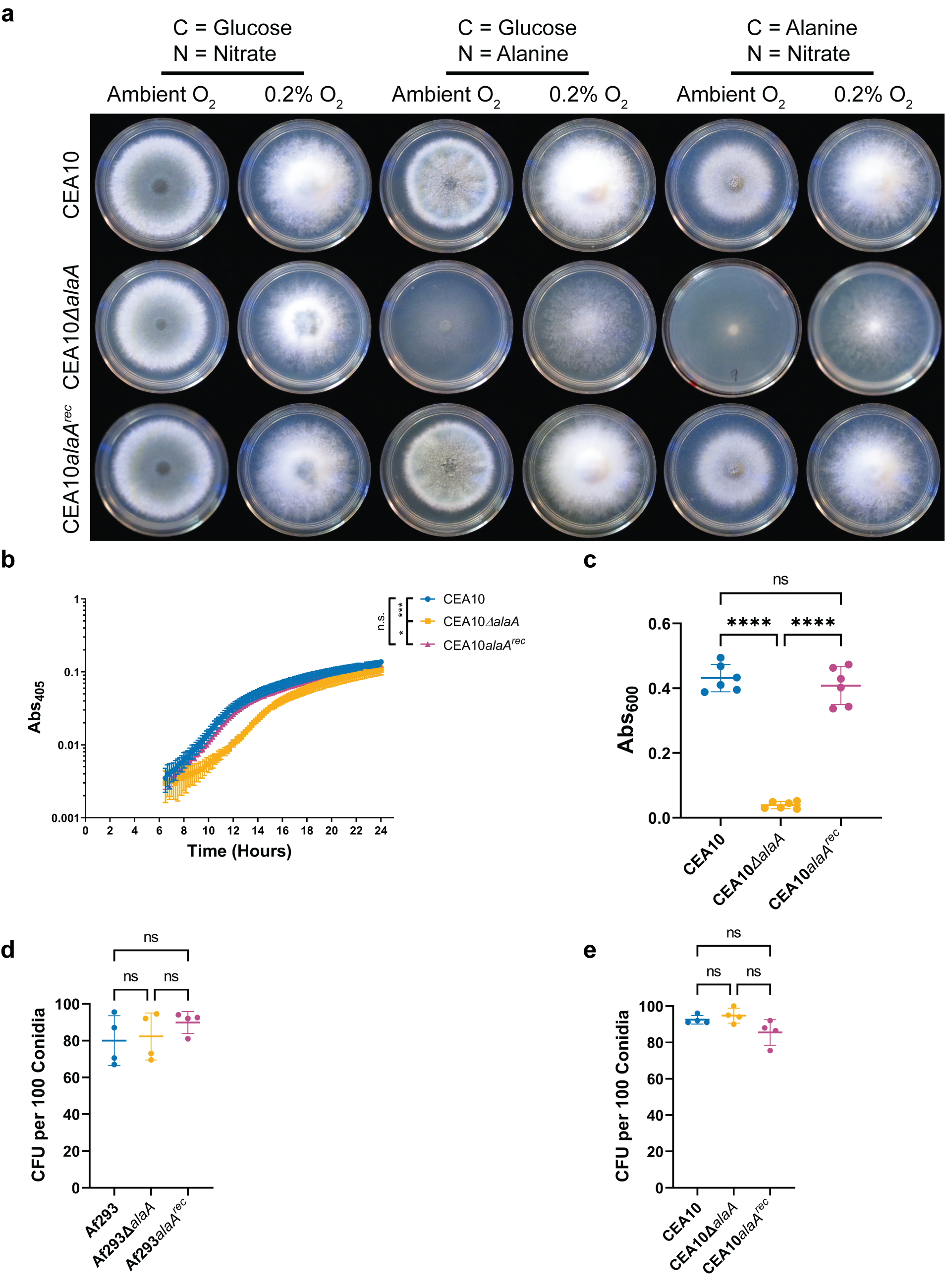

Supplement: FIG S1 [file mbio.02933-21-sf001.tif]

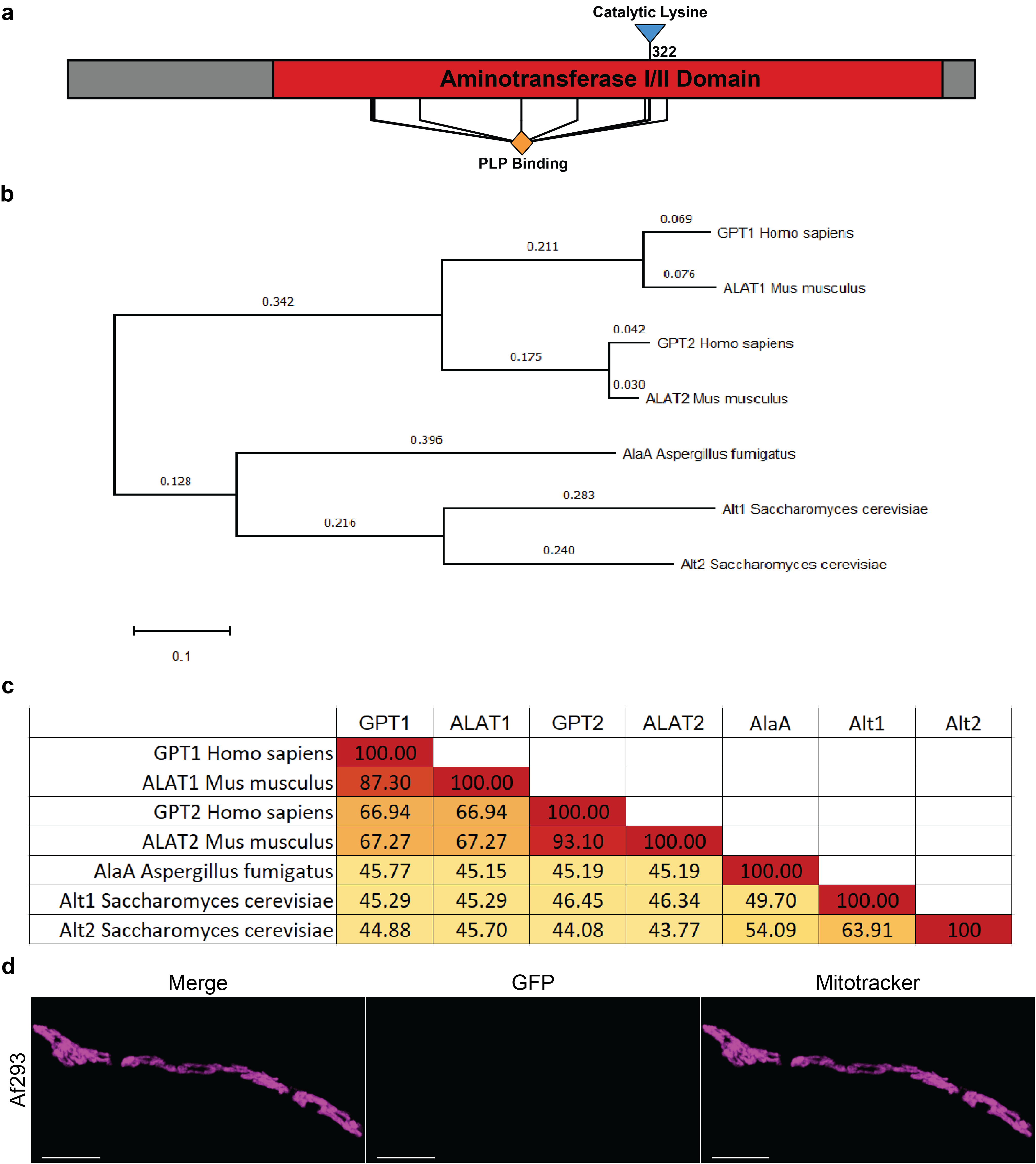

Supplement: FIG S2 [file mbio.02933-21-sf002.tif]

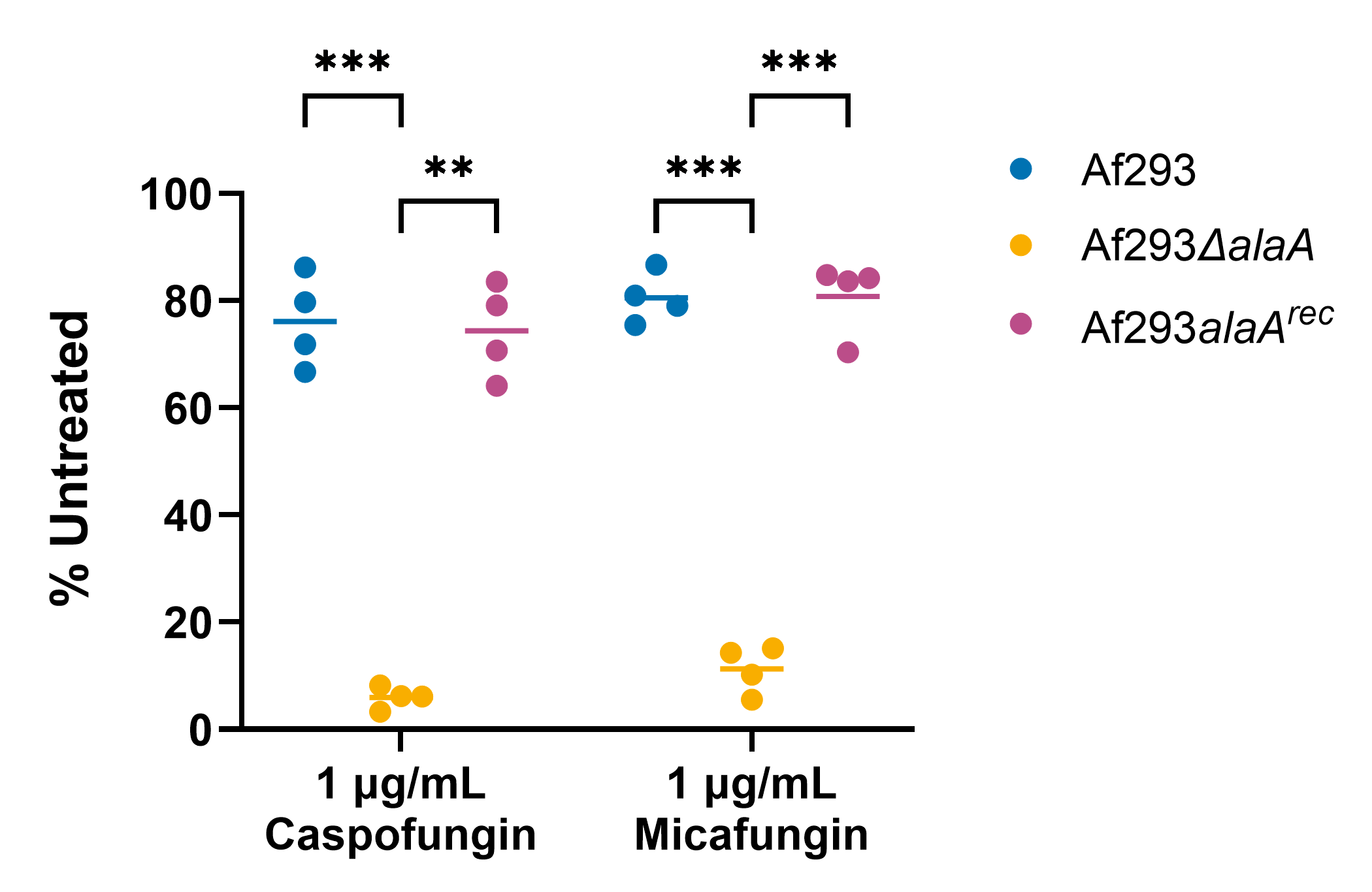

Supplement: FIG S3 [file mbio.02933-21-sf003.tif]

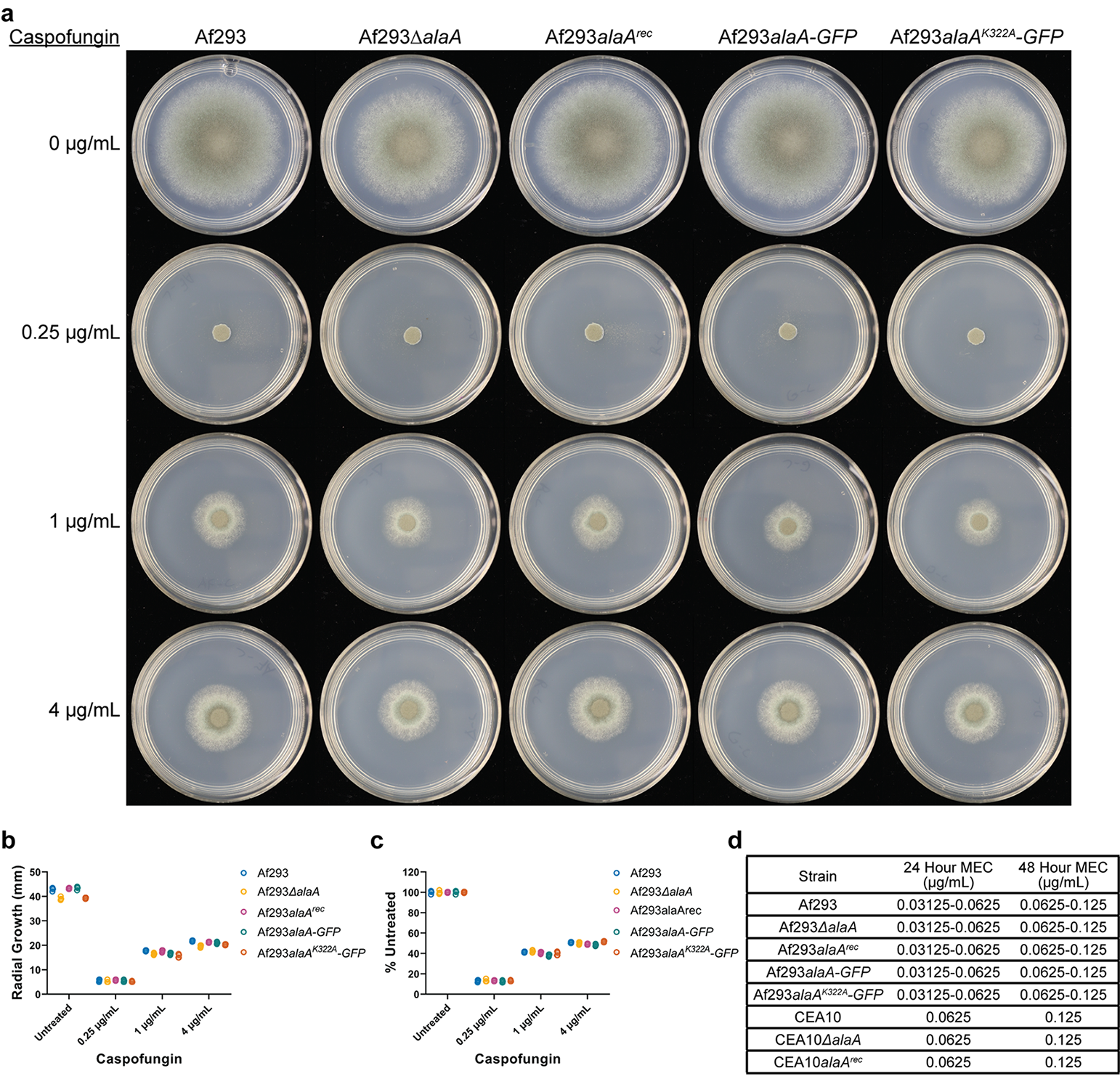

Supplement: FIG S4 [file mbio.02933-21-sf004.tif]

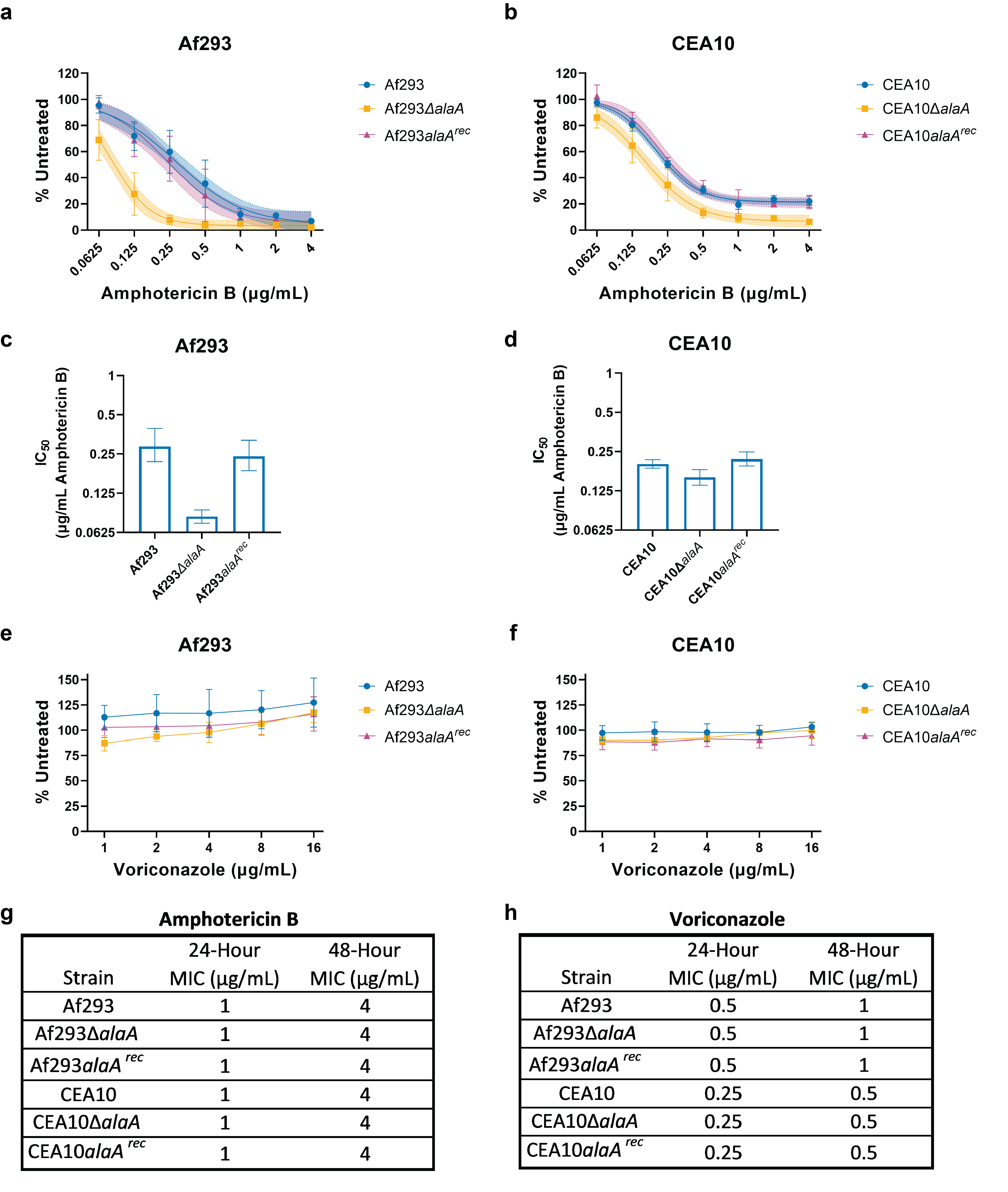

Supplement: FIG S5 [file mbio.02933-21-sf005.tif]

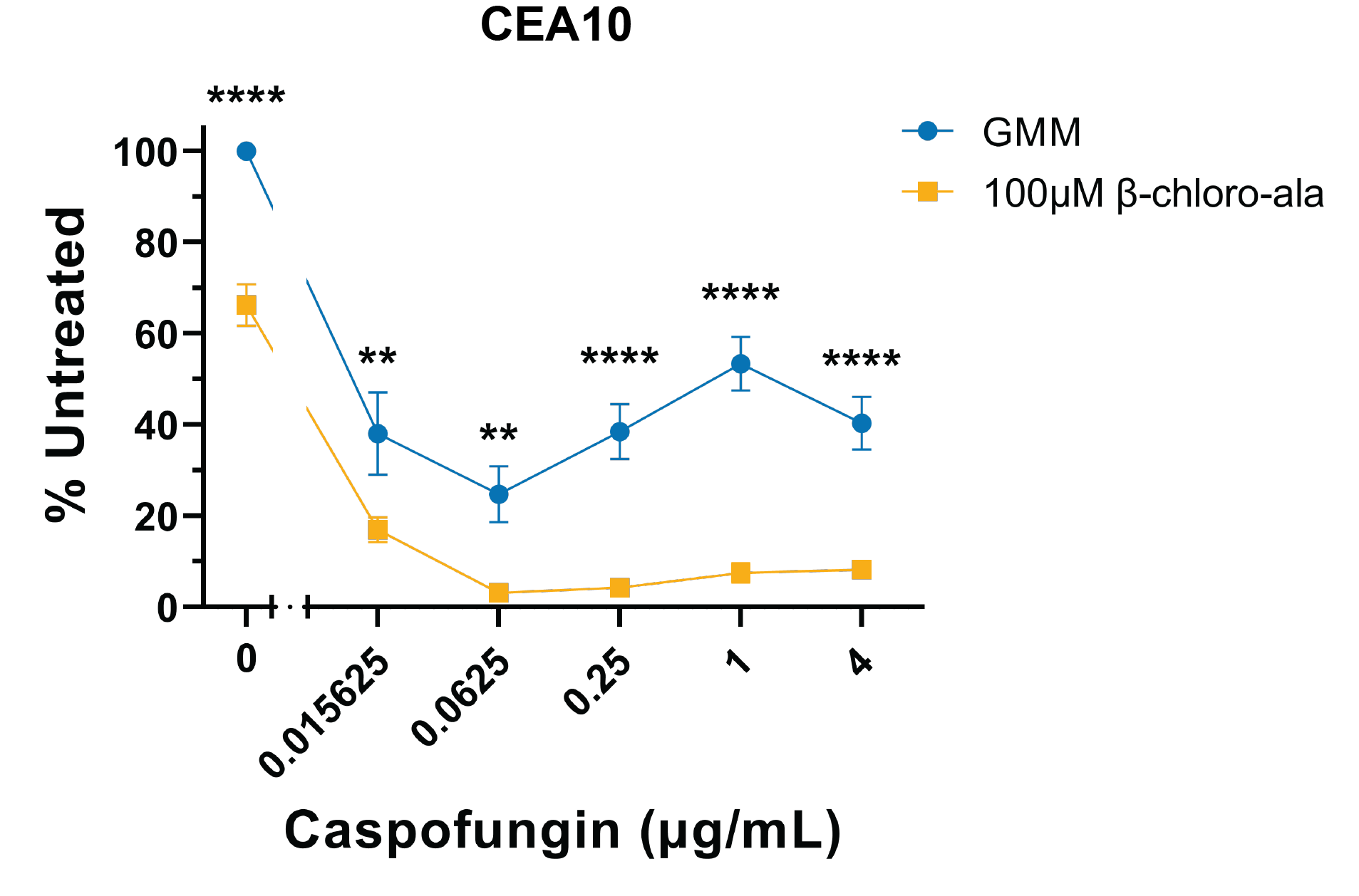

Supplement: FIG S6 [file mbio.02933-21-sf006.tif]

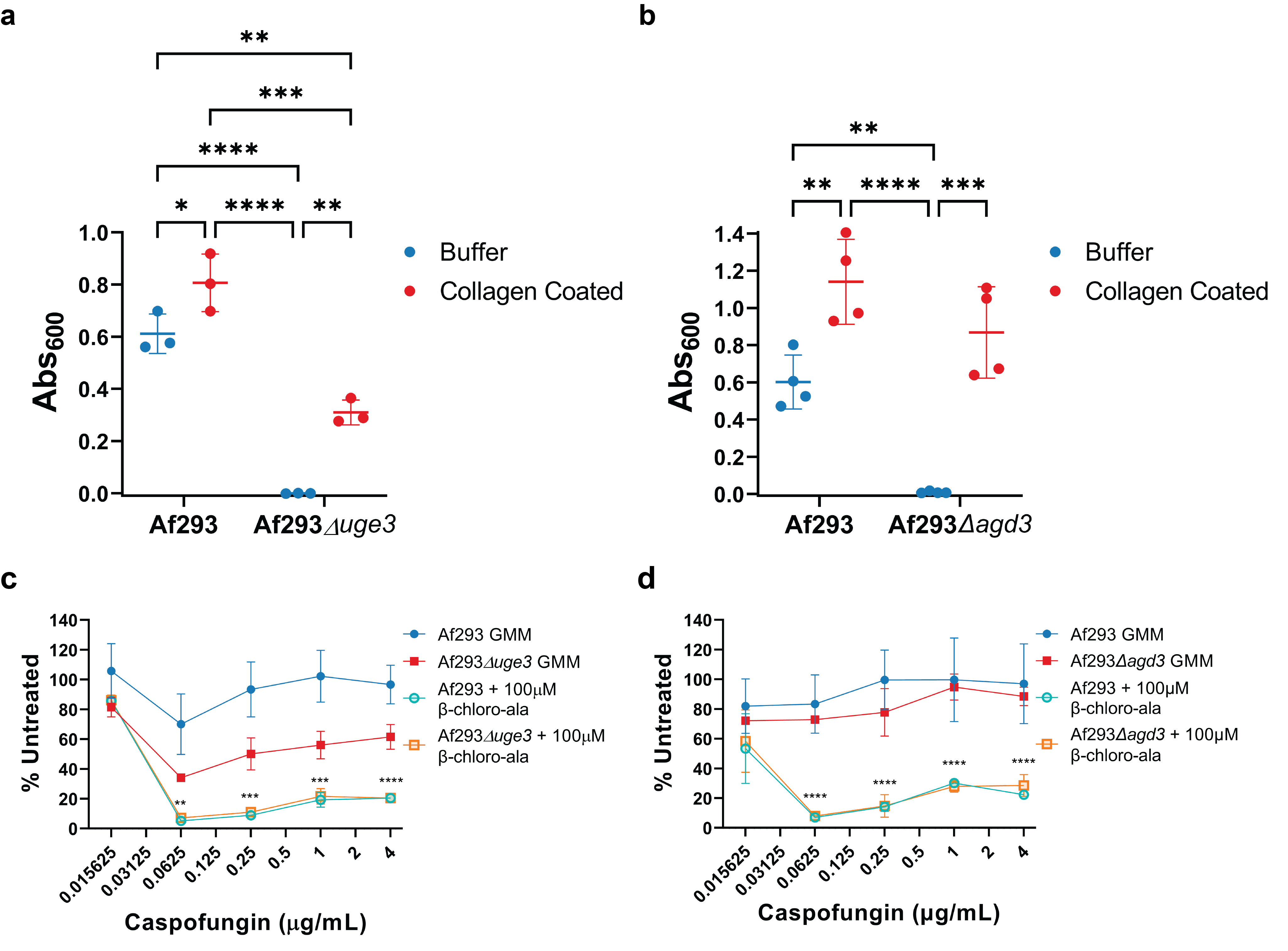

Supplement: FIG S7 [file mbio.02933-21-sf007.tif]

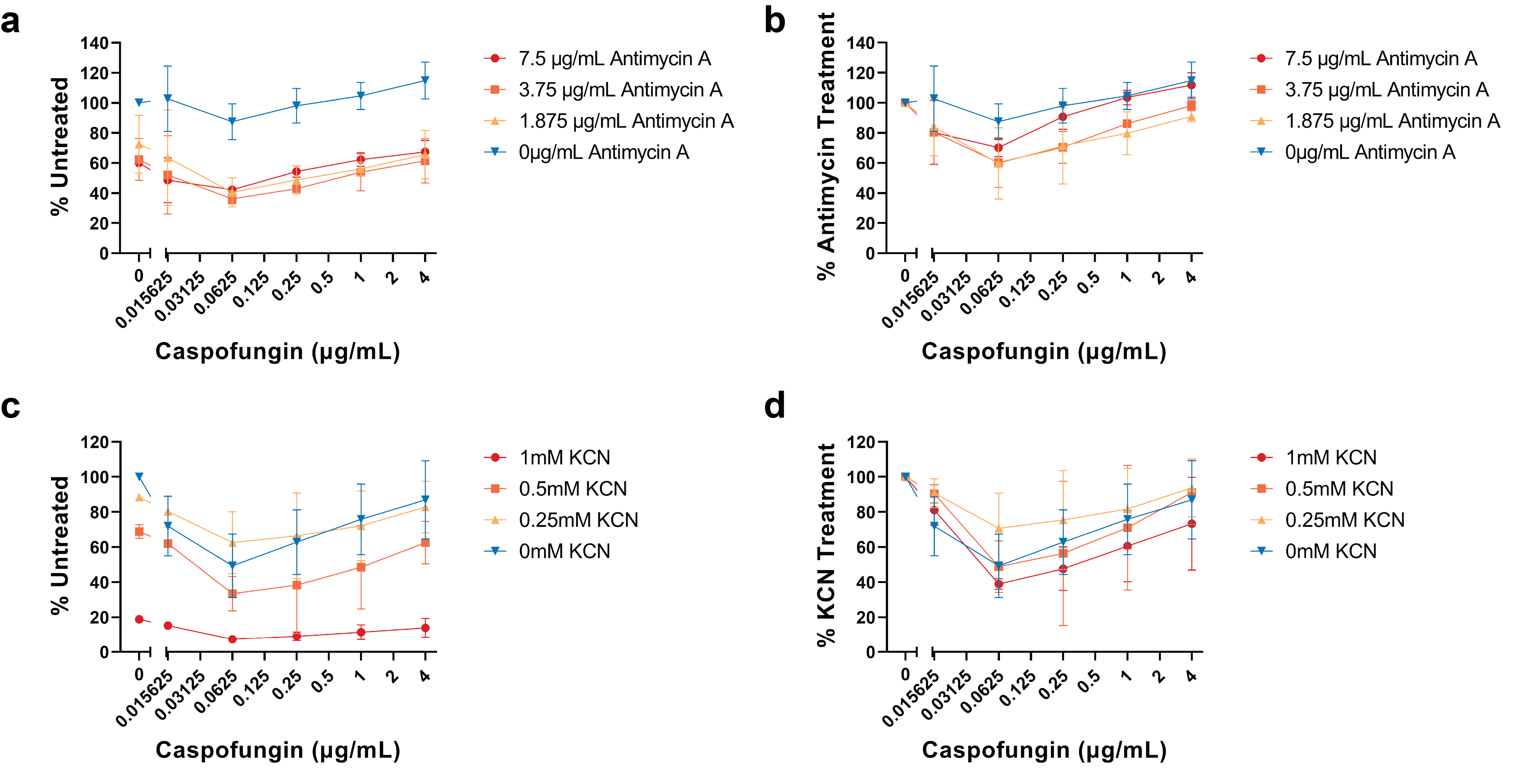

Supplement: FIG S8 [file mbio.02933-21-sf008.tif]

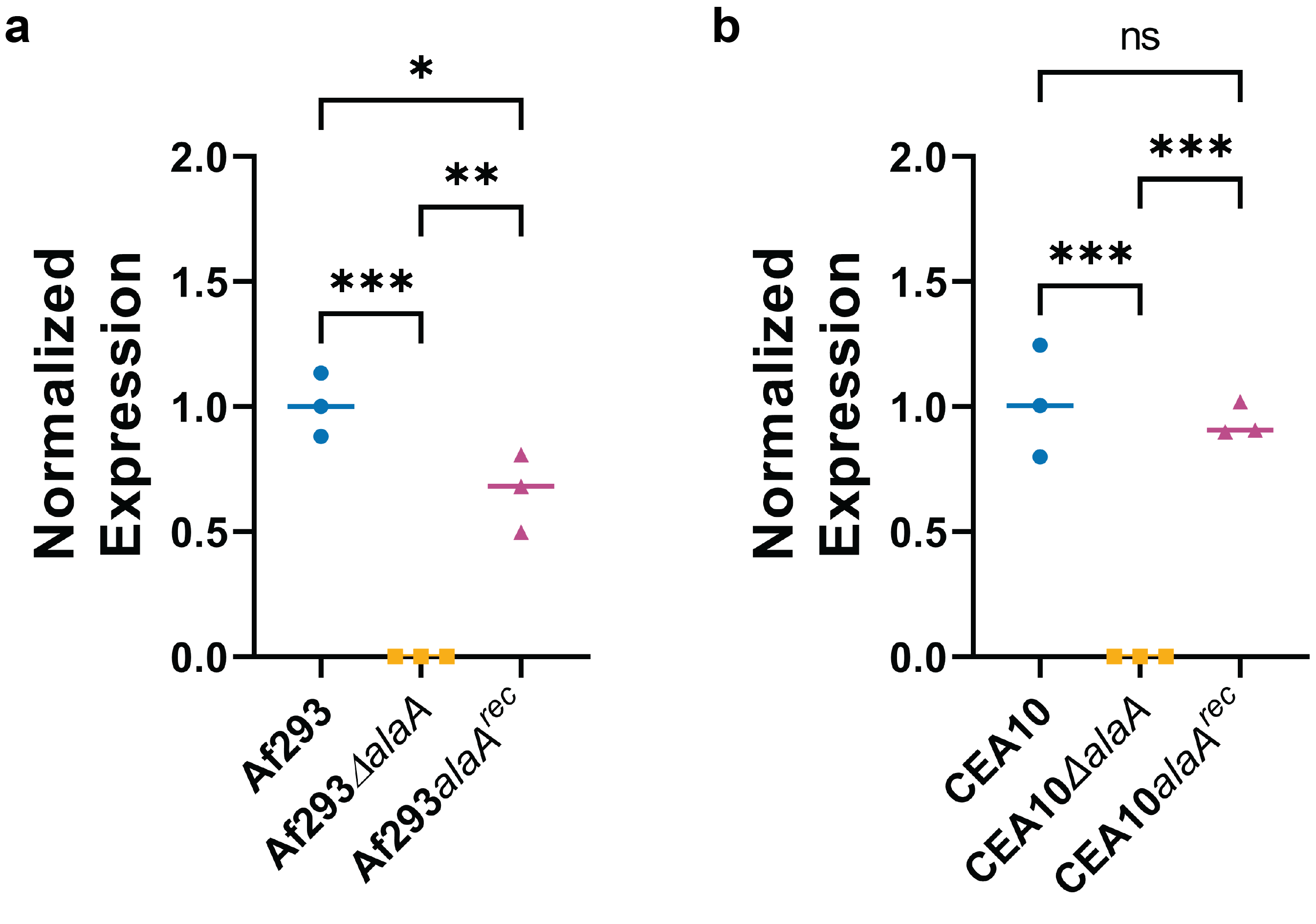

Supplement: FIG S9 [file mbio.02933-21-sf009.tif]
