# Supplementary material for: An Alanine Aminotransferase Is Required for Biofilm-Specific Resistance of Aspergillus fumigatus to Echinocandin Treatment
Source: mBio. 2022 Mar 7;13(2):e02933-21. doi: 10.1128/mbio.02933-21 (PMC9040767; doi:10.1128/mbio.02933-21)
Supplement: TABLE S1 [file mbio.02933-21-st001.docx]

**Table S1: Fungal strains used in this study.**

| Strain | Background Strain | Genotype | Origin |
| --- | --- | --- | --- |
| Af293 | Reference Strain | N/A | Nierman et al., 2005 |
| Af293Δ*alaA* | Af293 | Δ*alaA*; *ptrA*+ | This Study |
| Af293*alaA^rec^* | Af293Δ*alaA* | *alaA*+; *ptrA*+; *hygR+* | This Study |
| CEA10 | Reference Strain | N/A | Girardin et al., 1993 |
| CEA10Δ*alaA* | CEA10 | Δ*alaA*; *ptrA*+ | This Study |
| CEA10alaA^rec^ | CEA10Δ*alaA* | *alaA*+; *ptrA*+; *hygR+* | This Study |
| Af293*alaA-GFP* | Af293 | *alaA-GFP*; *ptrA*+ | This Study |
| Af293*alaA^K322A^-GFP* | Af293 | *alaA^K322A^-GFP*; *ptrA*+ | This Study |
| Af293Δ*agd3* | Af293 | Δ*agd3*; *hygR*+ | Lee et al., 2016 |
| Af293Δ*uge3* | Af293 | Δ*uge3*; *hygR*+ | Gravelat et al., 2013 |
